# Hematological consequences of environmental change during dewilding of rhesus macaques

**DOI:** 10.1038/s41467-026-75260-w

**Published:** 2026-07-10

**Authors:** Annabelle Smith, Kasalina Kiwanuka, Gabriela Pessenda, Andrew R. Rahmberg, Jacob K. Flynn, Richard Herbert, Jason M. Brenchley, P’ng Loke

**Affiliations:** 1 Type 2 Immunity Section, Laboratory of Parasitic Diseases, NIAID, NIH, Bethesda, MD USA; 2 Barrier Immunity Section, Lab of Viral Diseases, NIAID, NIH, Bethesda, MD USA; 3 Comparative Medicine Branch, NIAID, NIH, Bethesda, MD USA

**Keywords:** Haematopoiesis, Osteoimmunology, Neutrophils, Rhesus macaque

## Abstract

The environment shapes immune system development and the regulation of inflammatory responses, however the hematological consequences of a major environmental change, such as those experienced during migration, remain poorly understood. Here, we assess the immunological consequences in male rhesus macaques as they transitioned from an outdoor provisioned environment to an indoor laboratory facility in a process we term ‘dewilding.’ Dewilding decreased neutrophils and increased lymphocytes, skewing toward a T_H_1 response and increased T cell activation. In the gut microbiome, fungal abundance decreased while bacterial abundance increased. In the bone marrow, we observed a shift towards the less committed multipotent progenitor cells and increased erythrocyte progenitors, with upregulation of genes involved in hemoglobin control and erythropoiesis. Together, our findings illustrate how dewilding alters immune homeostasis, with implications for understanding immune adaptation in migrants from rural to urban environments and for optimizing immunization strategies during environmental change.

## Abstract (web summary)

Environmental factors shape the immune system, but it is difficult to standardise these effects due to diversity of the impact. Here the authors assess the immunological consequences of environmental switching from an outdoors enclosure to indoors laboratory conditions in male rhesus macaques.

## Introduction

The immune system can be shaped by environmental and lifestyle factors. Environmental drivers of immune traits include nutrition, aging, seasonal variation, and the microbiome. Migration and industrialization continue to introduce humans to environmental changes, with immunological consequences including the changing prevalence of immune mediated inflammatory diseases^1^. The “Hygiene Hypothesis”^2,3^ proposed that less hygienic environments helped protect against the development of atopy, while the “Old Friends Hypothesis”^4^ posited that microbes that co-evolved with humans are protective against a range of inflammatory diseases. More recently, the microbiome has been characterized as a regulator of the immune response, with less diverse microbiota composition, commonly found in westernized communities, contributing to inferior immune regulation^5^. Concurrently, decreased exposure to common microbial pathogens and commensals reduces the body’s efficiency at producing immune-regulating regulatory T (T_Reg_) cells^6–8^. This inefficiency increases the risk of allergic responses to common environmental antigens and decreases the body’s defense against autoimmune disease. However, continuous exposure to antigens and persistent challenges of the immune system can lead to naïve T cell depletion, heightened immune activation, and skewing of the immune system, and increased immunological aging that may affect responses to vaccines^9^. To better understand these complex interactions, systems immunology approaches have begun to decipher the relative contributions of environmental and genetic factors to the variation in the human immune system^10–12^.

Recently, we have performed immunological studies releasing laboratory mice into an enclosed, outdoor, enclosure, a process we call “re-wilding,” to examine genetic and environmental contributions to immune variation. Rewilded mice demonstrated greater gastrointestinal (GI) microbiome diversity (both prokaryotic and eukaryotic) and expanded granulopoiesis compared to laboratory mice^13^, and they became more susceptible to infection with the intestinal nematode parasite *Trichuris muris*^14^, which dramatically influences mucosal immunity and protection against bacterial infections^15^. The re-wilding-induced increase in granulocyte populations in the blood made them more “human-like,” as mouse blood usually has far fewer granulocytes than human blood. Fungal colonization was identified as a major driver of this increased granulopoieses^16^, and it conferred greater long-term protection from bloodstream infection by Gram-positive bacteria than by transient exposure to the fungal cell wall component β-glucan^13^. By flow cytometry profiling, we found that the cellular composition in both the peripheral blood and the mesenteric lymph nodes was shaped by interactions between genotype and environment^17^, which is consistent with some human studies^18,19^ and murine studies of pet shop and wild microbiome associations^20,21^. B cells also underwent major changes following rewilding, with increased signs of maturation and heightened germinal center responses within secondary lymphoid organs^22^. There were also expanded B cells in the GI tract, which was accompanied by elevated systemic levels of immunoglobulin G (IgG) and IgM antibodies reactive to the microbiota. Whereas variation in the expression of CD44 on T cells was driven mostly by genetics, expression of CD44 on B cells was explained more by the environment across different inbred strains of mice^17^. When we tracked individual mouse behavior with subcutaneous radio-frequency identification and associated it with their immune phenotype, we found that social association between mice was particularly predictive of similar memory T and B cell profiles and was more influential than sibling relationships or shared infection status^23^. Consistent with some observations in humans^24,25^, the more socially associated two individuals were, the more similar their immune phenotypes were, with this effect particularly strong for cellular composition but weaker for cytokines.

While rewilding may bring a laboratory mouse closer to the human immune system^26^, non-human primates are better for modeling human immunology, with the advantage of performing experimental interventions that are difficult in clinical studies^27,28^. Hence, we have developed a “dewilding” model transitioning rhesus macaques (*Macaca mulatta*) from a provisioned outdoor environment to indoor research facilities. Using this model, we have previously shown that transition to captivity has a large impact on the composition of the GI tract bacterial microbiome^29^. This transition mirrors human migration from rural developing regions to more industrialized urban environments, providing a unique opportunity to study the molecular and cellular effects of environmental changes on the immune system.

Here, we observe some parallels with previous rewilding projects in that neutrophils are decreased and lymphocytes increased in the peripheral blood after dewilding, which is the opposite of rewilding. However, T cell activation markers are increased in dewilding, just as they are increased in rewilding. Therefore, the effects of environmental change may have distinct influences on myeloid cells compared to T cell differentiation, which led us to further examine the bone marrow in dewilded macaques to investigate hematopoietic stem and progenitor cell (HSPC) populations. Our findings provide valuable insights into how environmental changes impact the immune system, revealing that dewilding distinctly influences the differentiation trajectory of hematopoietic stem cells and subsequently affects the populations of immune cells in the peripheral blood, notably T cells and granulocytes.

## Results

### Dewilding decreases peripheral granulocytes and increases peripheral lymphocytes

**Fig. 1:**
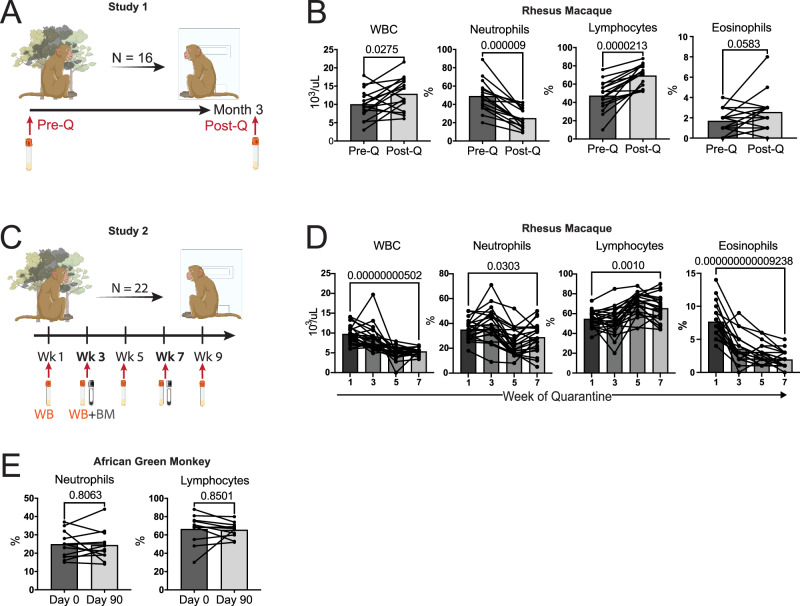
Dewilding leads to reduced neutrophils and increased lymphocytes. **A** Schematic of dewilding study 1 (*N* = 16) showing whole blood (WB) sampling of rhesus macaques for CBC (Fig. 1, in vitro stimulation (Fig. 2), and flow cytometry (Fig. 3) before (Pre-Q) and after 3-month quarantine in a lab environment (Post-Q). **B** Before-and-after bar plots showing the percentages of white blood cells (WBC), neutrophils, lymphocytes, and eosinophils measured in rhesus macaques before (Pre-Q) and after 3 months of quarantine in a lab environment (Post-Q), as assessed by complete blood count (CBC) with differential (*N* = 16). Effect sizes: WBC 0.284, neutrophils 0.742, lymphocytes 0.695, eosinophils 0.219. **C** Schematic of dewilding study 2 (*N* = 22) showing whole blood (WB) and bone marrow (BM) sampling of rhesus macaques for CBC (Fig. 1) and flow cytometry (Fig. 3) at weeks 1, 3, 5, 7, and 9 of the dewilding quarantine period. **D** Bar plots showing the concentration of WBC, neutrophils, lymphocytes, and eosinophils measured in rhesus macaques at weeks 1, 3, 5, and 7 of quarantine in a lab environment (Post-Q), as assessed by complete blood count (CBC) with differential (*N* = 22). Effect sizes: WBC 0.811, neutrophils 0.205, lymphocytes 0.410, eosinophils 0.895. **E** Before-and-after bar plots showing the percentages of neutrophils and lymphocytes measured in African green monkeys at day 0 and day 90 in a lab environment, as assessed by complete blood count (CBC) with differential. Effect sizes: AGM neutrophils 0.0057, AGM lymphocytes 0.0037. Points in bar plots represent individual rhesus macaques or African green monkeys. *P* values were determined by paired two-tailed *t*-test. Effect sizes for group differences were quantified using partial eta squared (ηp²). Graphics created in BioRender. Smith, A. (2026) https://BioRender.com/ftxte5e and Smith, A. (2026) https://BioRender.com/9td7gyk.

To determine the impact of “dewilding”—the transition from an outdoor environment to a controlled quarantine environment—on the immune system, we first analyzed blood samples from rhesus macaques (*N* = 16) before and after they completed a 3-month quarantine period after moving into an indoor facility (Fig. 1A). Complete blood counts (CBC) with differential analysis showed a significant reduction of neutrophils and expansion of lymphocytes in the peripheral blood (Fig. 1B). A second study (*N* = 22) was then conducted, whereby blood was collected at several time points during the dewilding period (Fig. 1C). Consistent with the first study, there was a significant reduction of neutrophils and increase of lymphocytes in the peripheral blood. However, in contrast to study 1, whereby dewilding slightly increased white blood cells (WBC) and eosinophils in the blood, in study 2 there was a sharp drop in WBCs and eosinophils during the dewilding period (Fig. 1D). Other hematological parameters were not consistent or significantly altered across both the studies, including data on monocyte percentage, hematocrit, hemoglobin concentration, or red blood cell count (Fig. S1). To determine if changes in neutrophils and lymphocytes were due to aging or the effects of extended time in the quarantine environment, we analyzed CBC with differential data of age-matched African green monkeys (AGMs) who were born in captivity, had never been in an outdoor facility, and had, thus, not undergone environmental change (Fig. 1E). These animals are natural hosts for SIVagm and a small colony was imported from Tanzania into our facility more than 20 years ago. To maintain this small colony, a small in house breeding colony was initiated^30,31^. AGMs did not exhibit any discernable changes in neutrophil or lymphocyte populations during a similar time.

Overall, these results indicate that there are consistent alterations in the peripheral blood neutrophil and lymphocyte compartments in dewilded macaques, whereas changes in total WBCs and eosinophils varied between the two experiments (potentially due to decreased parasite burden following a deworming procedure during the transition^29^). While data from cohort 1 was unavailable, we determine that 100% of the animals from cohort 2 (*N* = 22) were positive for *Trichuris* parasites and 59% (*N* = 13) were positive for *Strongyloides* in fecal samples. Serology analysis from the past indicated that 25.5% (*N* = 12/47) of rhesus macaques were seropositive for *Strongyloides*.

### Dewilding shifts a T_H_17 response to a TH1 response and increases activation and maturation of T cells

To investigate how dewilding affects cytokine responses of immune cells in the blood we stimulated the whole blood of animals from study 1 with different antigens (*Staphylococcus aureus* (SA), *Pseudomonas aeruginosa* (PA), *Bacillus subtilis* (BS), House Dust Mite (HDM), *Clostridium perfringens* (CP), *Bacteroides vulgatus* (BV) and *Candida albicans* (CA)) and profiled the production of cytokines (IL-23, IL-12p40, CXCL10, IL-6, IL-10, IL-8, IL-1β, IFN-γ, CCL2, IFN-β, TNF-α, GM-CSF, and IL-17) using a multiplex cytokine array (Fig. S2).

**Fig. 2:**
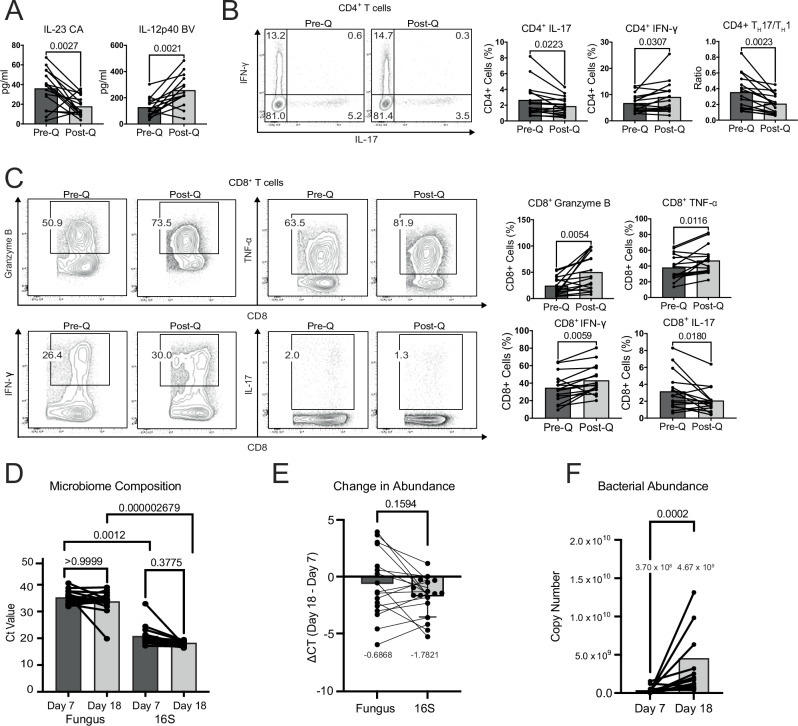
Dewilding-driven shifts in pathogen-specific immune responses and T-cell cytokine production. **A** Before-and-after bar plots showing the cytokine response to in vitro stimulation by *Candida albicans* (T_H_17) or *Bacteroides vulgatus* (T_H_1), as demonstrated by the concentration of IL-23 (Effect size: 0.463) or IL-12p40 (Effect size: 0.531), respectively, before and after quarantine (*N* = 16). **B** Representative flow cytometry analysis and quantification of intracellular cytokine staining for IFN- γ (Effect size: 0.275) and IL-17 (Effect size: 0.302) in CD4^+^ T cells, including quantification of CD4^+^ T_H_17/T_H_1 response ratio (Effect size: 0.471), before and after quarantine (*N* = 16). **C** Representative flow cytometry analysis and quantification of intracellular cytokine staining for Granzyme B (Effect size: 0.413), TNF-α (Effect size: 0.356), IFN- γ (Effect size: 0.406), and IL-17 (Effect size: 0.320) in CD8^+^ T cells, before and after quarantine. **D** Before-and-after bar plots of Cycle Threshold (Ct) values from quantitative PCR (qPCR) measuring abundance of fungus and bacteria at day 7 and day 18 of dewilding (*N* = 17). Statistical analyses shown are Dunn’s multiple comparisons test. Effect was summarized using Friedman statistics and one-sided Wilcoxon signed‑rank tests (Friedman *χ*² = 42.18 for all within‑subject microbiome comparisons; Wilcoxon *W* = −61 for change in abundance and *W* = 141 for bacterial abundance). **E** Before-and-after bar plots showing the change in abundance of fungus and bacteria in macaque stool during dewilding, calculated as the ΔCt = Ct_day18_ − Ct_day7_ (*N* = 17). **F** Before-and-after bar plots showing the bacterial copy number in macaque stool at day 7 and day 18 of dewilding (*N* = 17). Points in bar plots represent individual rhesus macaques. *P* values were determined by paired two-tailed *t*-test unless otherwise stated. Effect sizes for cytokine and cytotoxic responses were quantified using partial eta squared (ηp²).

Notably, after dewilding, the IL-23 response to CA was significantly reduced and the IL-12p40 response to BV was significantly increased (Fig. 2A). Additionally, other Type 1-associated cytokines including CXCL10 (in response to SA and CP), TNF-α (in response to PA), and IFN-γ (in response to BS) were increased following dewilding, suggesting a shift toward a Type 1 response after quarantine. Type 2-associated cytokines were not altered by dewilding (Fig. S2).

We next examined the capacity of CD4+ and CD8+ T cells to produce cytokines after phorbol myristate acetate (PMA) and ionomycin stimulation, by intracellular cytokine staining of peripheral blood mononuclear cells (PBMCs) (Figs. S3–4). In CD4^+^ T cells, there was a significant reduction of IL-17^+^ cells and a significant increase of IFN-γ^+^ cells after dewilding (Fig. 2B). Thus, the ratio of CD4^+^ T_H_17/T_H_1 cells is decreased after dewilding (Fig. 2B). For CD8^+^ T cells, following dewilding, there were also significantly more Granzyme B, TNF-α, and IFN-γ positive CD8^+^ T cells (Fig. 2C). While the numbers are very low, there were fewer IL-17 positive CD8^+^ T cells after dewilding (Fig. 2C). Foxp3^+^ T_Reg_ populations were unaltered by dewilding (Fig. S5). Apart from a slight reduction in IL-5^+^ CD4^+^ and CD8^+^ T cells and in IL-2^+^ CD8^+^ T cells, there were no other changes to cytokine positive T cells following dewilding (Fig. S5). Overall, these results indicate a dewilding-induced shift from a Type 17 response to a Type 1 response, which is consistent with the reduction in neutrophil levels in the blood.

To determine if fungal and bacterial abundances in stool samples were altered during dewilding, we analyzed the DNA extracted from stool samples collected longitudinally during the dewilding process. Quantitative PCR (qPCR) analysis of fungal DNA and bacterial DNA was performed to calculate fungal and bacterial Cycle Threshold (Ct) values and bacterial copy number^32^. We calculated difference in Ct values (ΔCt = Ct_day18_ − Ct_day7_) for fungal and bacterial DNA to determine changes in abundance during dewilding. As expected, fungal DNA quantities were consistently lower than bacterial DNA across all samples (Fig. 2D). Notably, during dewilding, there is increasing abundance of bacterial copy numbers (Fig. 2E), whereas fungal abundance remains the same. Bacterial copy number significantly increased, more than 12-fold during dewilding (Fig. 2F); hence while fungal DNA levels remained relatively stable, bacterial density in the stool increased during dewilding. However, these results lack taxonomic resolution without metagenomic sequencing of the microbiome, which was not feasible in this study.

**Fig. 3:**
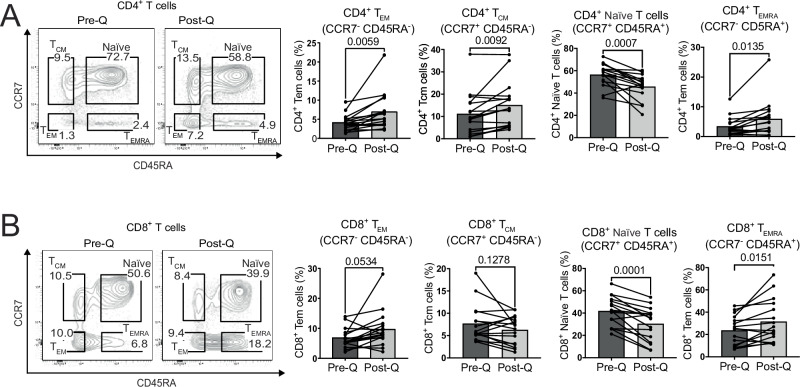
Dewilding-driven shifts in T-cell populations. Representative flow cytometry analysis and quantification of central memory (T_CM_), effector memory (T_EM_), and naïve T cells for CD4^+^ (**A**) and CD8^+^ (**B**) T cells, before and after quarantine (*N* = 16). Effect sizes: CD4⁺ T_EM_ (CCR7⁻ CD45RA⁺) pre‑Q vs post‑Q = 0.406, CD4⁺ T_CM_ (CCR7⁺ CD45RA⁻) = 0.373, CD4⁺ naïve T cells (CCR7⁺ CD45RA⁺) = 0.544, CD4⁺ T_EMRA_ (CCR7⁻ CD45RA⁺) = 0.343; CD8⁺ T_EM_ (CCR7⁻ CD45RA⁻) = 0.335, CD8⁺ T_CM_ (CCR7⁺ CD45RA⁻) = 0.148, CD8⁺ naïve T cells (CCR7⁺ CD45RA⁺) = 0.636, CD8⁺ T_EMRA_ (CCR7⁻ CD45RA⁺) = 0.227. Points in bar plots represent individual rhesus macaques. *P* values were determined by paired two-tailed *t*-test. Effect sizes for T‑cell subset changes were quantified using partial eta squared (ηp²).

We previously found that during rewilding, mice increase T memory (T_EM_) and T central memory (T_CM_) cells while reducing naïve T cells in the blood^16,33^. Here, we find that dewilding macaques also increases CD4^+^ effector and central memory T cells^34^, while naïve T cells were reduced after dewilding (Fig. 3A). CD8^+^ effector memory T cells were also increased during dewilding, and naïve T cells were reduced following dewilding (Fig. 3B). However, no significant changes were observed in the population of CD8^+^ central memory T cells. Consequently, while some aspects of dewilding are consistent with reversing the effects of rewilding (i.e., neutrophil populations in the blood), other immune features of rewilding (i.e., T cell maturation/activation) were not altered by dewilding, indicating that both types of environmental changes mature T cell populations in the blood overall.

### Dewilding induces distinct changes to hematopoietic precursor cells in the bone marrow

**Fig. 4:**
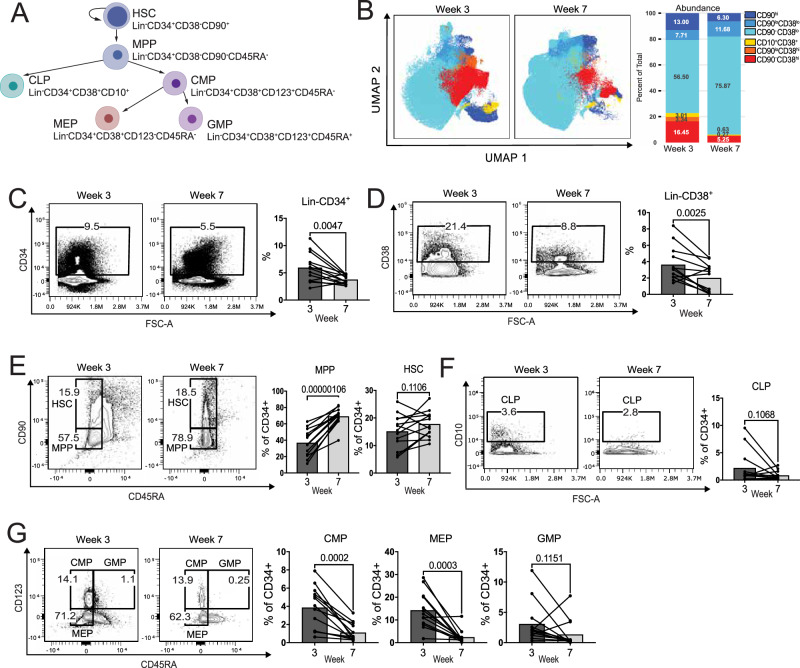
Dewilding-driven shifts in hematopoietic compartment and progenitors in the bone marrow. **A** Schematic of hematopoietic stem and progenitor cell populations and their distinctive cellular markers to be assessed by flow cytometry. **B** UMAP visualization showing the changes in hematopoietic stem cell (HSC) populations based on CD90 and CD38 expression, derived from flow cytometry analysis of bone marrow cells from dewilded rhesus macaques, observed at week 3 and week 7 of dewilding (*N* = 13). **C** Representative flow cytometry analysis and quantification of CD34^+^ hematopoietic stem cell compartment at week 3 and week 7 of dewilding (*N* = 13). Effect size = 0.499. **D** Representative flow cytometry analysis and quantification of CD38^+^ hematopoietic progenitor cell compartment at week 3 and week 7 of dewilding (*N* = 13). Effect size = 0.547. **E** Representative flow cytometry analysis and quantification of CD34^+^CD38^-^CD90^+^CD45RA^-^ hematopoietic stem cells (HSC) and CD34^+^CD38^-^CD90^-^CD45RA^-^ multipotent progenitor (MPP) cells at week 3 and week 7 of dewilding (*N* = 13). Effect size = 0.872. **F** Representative flow cytometry analysis and quantification of CD34^+^CD38^+^CD10^+^ common lymphoid progenitor (CLP) cells at week 3 and week 7 of dewilding (*N* = 13). Effect size for multipotent progenitors (MPP) = 0.872, hematopoietic stem cells (HSC) = 0.198, common lymphoid progenitors (CLP) = 0.202. **G** Representative flow cytometry analysis and quantification of Lin^-^CD34^+^CD38^+^CD123^+^CD45RA^-^ common myeloid progenitor (CMP), Lin^-^CD34^+^CD38^+^CD123^-^CD45RA^-^ megakaryocyte-erythroid progenitor (MEP), and Lineage ^-^CD34^+^CD38^+^CD123^+^CD45RA^+^ granulocyte-monocyte progenitor (GMP), at week 3 and week 7 of dewilding (*N* = 13). Effect size for common myeloid progenitors (CMP) = 0.698, megakaryocyte–erythroid progenitors (MEP) = 0.686, granulocyte–monocyte progenitors (GMP) = 0.194. Points in bar plots represent individual rhesus macaques. *P* values were determined by paired two-tailed *t*-test. Effect sizes for hematopoietic populations were quantified using partial eta squared (ηp²).

Rewilding laboratory mice can enhance granulopoiesis in the bone marrow (BM)^13^. Thus, we collected BM samples from the macaques during the dewilding period (Fig. 1C) for flow cytometry analysis (Figs. S6–7). We focused our analysis on samples collected in the first and second month of dewilding, in weeks 3 and 7, where major changes in the blood were observed by CBC with differential analysis (Fig. 1D). We first investigated hematopoietic stem and progenitor cells (HSPCs) by analyzing CD34^+^ cells along with several differentiation and progenitor markers (CD10, CD38, CD45RA, CD90, CD123; Fig. 4A, Figs. S6A, S7–8).

Dimensionality reduction of the (13-color panel; Fig. S6A) flow cytometry data comparing week 3 to week 7 of dewilding revealed a reduction in the progenitor marker CD38 and in hematopoietic stem cell (HSC) marker CD90 expression within the CD34^+^ hematopoietic compartment (Fig. 4B). The frequency of lineage negative CD34^+^ and CD38^+^ cells are reduced during dewilding (Fig. 4C, D). Within the CD34^+^ hematopoietic compartment, the percentage of multipotent progenitor (MPP) cells was significantly increased (Fig. 4E). Although common lymphoid progenitor (CLP) and granulocyte-monocyte progenitor (GMP) cells were not significantly reduced (Fig. 4F, G), we observed a significant reduction in the megakaryocyte-erythrocyte progenitor (MEP) and common myeloid progenitor (CMP) (Fig. 4G). Overall, there is a shift from more committed progenitors, such as the CMP cells, towards the less committed MPP cells.

**Fig. 5:**
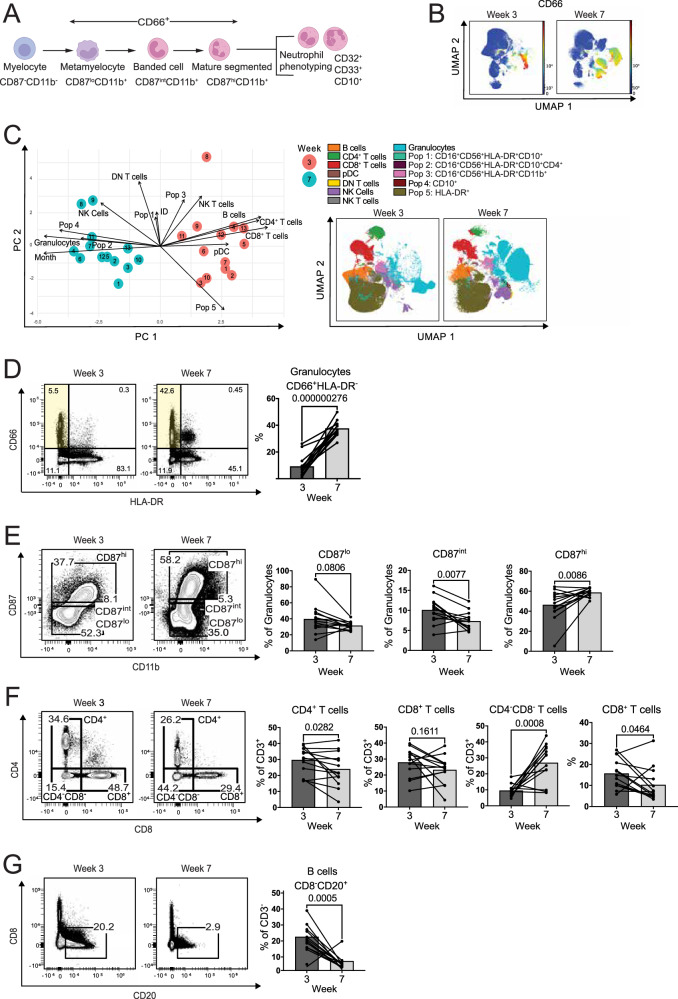
Dewilding-driven shifts in granulocytes and lymphocytes in the bone marrow. **A** Schematic of granulocyte progenitor and phenotypic populations and their distinctive cellular markers to be assessed by flow cytometry. Created in BioRender. Smith, A. (2026) https://BioRender.com/8laiyzf. **B** Uniform Manifold Approximation and Projection (UMAP) visualization of the change in CD66 expression in flow cytometry data from bone marrow cells of dewilded rhesus macaques, at week 3 and week 7 of dewilding (*N* = 13). **C** Principal component analysis (PCA) and UMAP visualization of immune cell populations identified with flow cytometry data from bone marrow cells of dewilded rhesus macaques, at week 3 and week 7 of dewilding (*N* = 13). **D** Representative flow cytometry analysis and quantification of CD66^+^HLA-DR^-^ granulocytes, at week 3 and week 7 of dewilding (*N* = 13). Effect size = 0.897. **E** Representative flow cytometry analysis and quantification of neutrophil maturation, quantifying CD87^low^CD11b^+^ metamyelocytes, CD87^int^CD11b^+^ banded neutrophils, and CD87^hi^CD11b^+^ mature segmented neutrophils, at week 3 and week 7 of dewilding (*N* = 13). Effect size for CD87^low^ granulocytes = 0.233, CD87^int^ granulocytes = 0.460, CD87^hi^ granulocytes = 0.450. **F** Representative flow cytometry analysis and quantification of CD4 and CD8 T cells, at week 3 and week 7 of dewilding (*N* = 13). Effect sizes for CD4⁺ T cells (% of CD3⁺) = 0.342, CD8⁺ T cells (% of CD3⁺) = 0.157, CD4⁺CD8⁺ T cells (% of CD3⁺) = 0.622, total CD3⁺ T cells = 0.291. **G** Representative flow cytometry analysis and quantification of CD8^-^CD20⁺ B cells, at week 3 and week 7 of dewilding (*N* = 13). Effect size = 0.651. Points in bar plots represent individual rhesus macaques. *P* values were determined by paired two-tailed *t*-test. Effect sizes for granulocyte and lymphocyte subsets were quantified using partial eta squared (ηp²).

We next investigated the CD34^-^ population, focusing on neutrophil maturity markers and phenotypes in the BM (Fig. 5A, Figs. S6B, S9–11). Uniform Manifold Approximation and Projection (UMAP) dimensionality reduction of (18-color panel, Fig. S6B) flow cytometry data revealed an increase in the frequency of CD66^+^ granulocytes within the CD34^-^ population (Fig. 5B), which contrasts with reduced neutrophil abundance observed in the blood. Principal component analysis (PCA) of the flow cytometry data (Fig. 5C) clearly demonstrated separation of the week 3 and week 7 BM samples. PCA and UMAP analyses established that granulocytes, CD4^+^ T-cell, CD8^+^ T-cell, and B-cell populations contributed greatly to this variation between week 3 and week 7 BM samples during dewilding. UMAP dimensionality reduction confirmed that after dewilding, there were dramatically higher numbers of granulocytes, while the CD4^+^ T-cell, CD8 + T-cell, and B-cell populations were reduced after dewilding (Fig. S6B).

We next confirmed by manual gating that there was a substantial increase in CD66^+^HLA-DR^-^ granulocytes in the BM during dewilding (Fig. 5D). In phenotyping this granulocyte population during dewilding, the CD87^int^CD11b^+^ banded cells were reduced, while the CD87^hi^CD11b^+^ mature segmented neutrophils were increased (Fig. 5E). CD32 expression was significantly reduced and CD10 expression was significantly increased in granulocytes during dewilding (Fig. S12A, D). We did not observe distinct changes in the other neutrophil markers we assessed. (Fig. S12B, C). In contrast, lymphocyte populations in the BM were reduced during dewilding (Fig. 5F, G). Specifically, the overall CD3^+^ T-cell compartment, CD4^+^ T cells, and B cells were significantly reduced in the BM. CD8^+^ T cells were also reduced during dewilding, although this reduction was not statistically significant. However, double-negative T cells were notably increased during dewilding. We also observed significant changes in other immune cell populations. Following dewilding, there was an increase in the mDC to pDC ratio (Fig. S12E) and reductions in non-classical monocytes, natural killer (NK) cells, and NK T cells (Fig. S12F–I).

Overall, the BM results indicate that cellular changes in the blood observed during dewilding are not directly reflected in changes to the composition of the BM (Fig. S13). More complex trafficking and retention mechanisms are at play contributing to changes in the hematological cells in the peripheral blood and BM during dewilding.

### Dewilding induces increased erythropoiesis

**Fig. 6:**
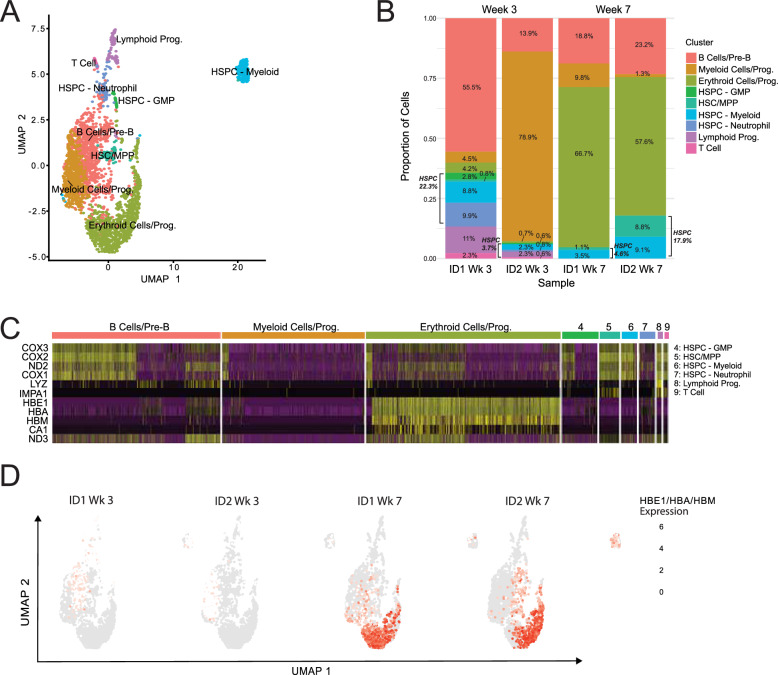
Dewilding-driven changes in cell populations and gene expression identified by single-cell nuclear RNA-Seq in the bone marrow. **A** Uniform Manifold Approximation and Projection (UMAP) visualization of the cell populations in the bone marrow identified by single-cell nuclear RNA-Seq, pooling samples from two representative animals at weeks 3 and 7 of dewilding (*N* = 4, *N* = 706 cells). **B** Composition plot of bone marrow cell populations identified by single-cell nuclear RNA-Seq, showing relative abundance changes at week 3 and week 7 of dewilding (*N* = 4). **C** Heatmap of differentially expressed genes across cell populations identified by single-cell nuclear RNA-Seq in bone marrow, highlighting distinct gene expression profiles primarily enriched in B Cells/Pre-B cells and erythrocytes (*N* = 4). **D** UMAP visualization of the expression of hemoglobin-associated genes HBE1, HBA, and HBM in the bone marrow at week 3 and week 7 of dewilding (*N* = 4, *N* = 706 cells).

To further investigate the transcriptional profile and composition of BM cells during dewilding, we analyzed single-cell nuclear RNA-Seq data from BM samples collected at weeks 3 and 7 (Fig. 1C). We identified nine distinct cell clusters and characterized their respective immune cell populations (Fig. 6A). The identified immune cell populations included: T Cells, Lymphoid Progenitors, Myeloid Hematopoietic Stem and Progenitor Cells, Neutrophil HSPC, Granulocyte Monocyte Progenitor (GMP) HSPC, B Cells/Pre-B, Hematopoietic Stem Cell (HSC)/Multipotent Progenitor (MPP), Myeloid Cells/Progenitors, and Erythroid Cells/Progenitors. Quantifying the changes in cluster proportions revealed a significant increase in erythroid cells and progenitors during dewilding (Fig. 6B). In Animal ID1, we observed a concurrent reduction in B cells, while in Animal ID2, there was a reduction in myeloid cells and progenitors. No distinct changes were observed in the proportions of hematopoietic stem and progenitor cells. Although their proportions were low, lymphoid progenitors and T cells were reduced during dewilding.

We assessed differentially expressed genes within each cluster and noted significant changes in genes related to cyclooxygenase enzymes (COX1-3) and hemoglobin regulation (HBA, HBE1, HBM) (Fig. 6C). Cyclooxygenase enzymes were particularly enriched in the following clusters: B Cells/Pre-B, HSC/MPP, HSPC - Myeloid, Lymphoid Progenitors, and T Cells. Genes associated with hemoglobin regulation were enriched in erythroid cells and progenitors, with their expression significantly increasing during dewilding (Fig. 6D). Overall, our results highlight distinct changes to BM immune cell populations during dewilding, notably an increase in erythroid cells and progenitors, indicating increased erythropoiesis during dewilding.

### Dewilding influences vaccine responses

**Fig. 7:**
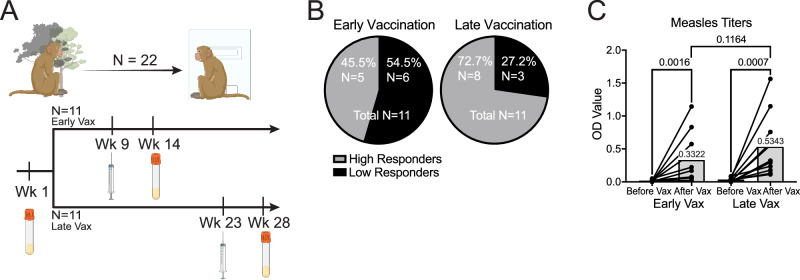
Changes to measles vaccine response given timing of vaccine during dewilding. **A** Schematic of the vaccination and serum sampling schedule, illustrating the differentiation between early (*N* = 11) and late (*N* = 11) vaccination groups based on the timing of vaccination during the dewilding process. Graphics created in BioRender. Smith, A. (2026) https://BioRender.com/ftxte5e and Smith, A. (2026) https://BioRender.com/9td7gyk. **B** Pie chart showing the percentage of responders and nonresponders to measles vaccination within the early (*N* = 11) and late (*N* = 11) vaccination groups. **C** Before-and-after bar plots of optical density values from enzyme-linked immunosorbent assay (ELISA) measurements of measles titers pre- and post-vaccination in early (*N* = 11) and late (*N* = 11) vaccination groups. Effect size for paired comparisons in (**C**) was estimated with epsilon‑squared (ε²) from the Kruskal–Wallis test; all three pairwise contrasts (Early pre‑vaccine vs Early post‑vaccine, Late pre‑vaccine vs Late post‑vaccine, Early post‑vaccine vs Late post‑vaccine) showed an effect of ε² KW ≈ 0.68. Statistical analyses shown are Kruskal-Wallis tests for pre- and post-vaccination comparisons within each group and Mann-Whitney test for post-vaccination comparison between early and late vaccination groups. *P* values were determined by Kruskal-Wallis test for comparisons within the vaccination groups and by two-tailed Mann-Whitney test for comparing the “After Vax” timepoint between early and late vaccination groups.

There is considerable geographic variation in vaccine responses between high-income and low-income countries, which could be driven by environmental factors^9^. To investigate if vaccine responses during dewilding is altered, measles vaccinations were administered to two groups of rhesus macaques at different timepoints during dewilding, with serum samples collected five weeks post-vaccination (Fig. 7A). Approximate measles titers based on optical densities (OD) were measured by enzyme-linked immunosorbent assays (ELISA) on serum samples before and after vaccination. While all the animals seroconverted, some had higher responses (>) and others had lower responses (<).

We noted that among the early vaccination group, there was an approximately equal distribution of high responders (*N* = 5) and low-responders (*N* = 6), whereas the late vaccination group showed a majority of macaques being high responders (*N* = 8) (Fig. 7B). However, these differences were not statistically significant by Fisher’s exact test (*P* = 0.38) or by a linear regression model in which the change in OD was regressed on vaccination groups (*R*² = 0.052, *P* = 0.137), likely because the experiment is underpowered. Although the post-vaccination OD value was higher in the late vaccination group (0.5343) compared to the early vaccination group (0.3322), this difference was also not statistically significant (*p* = 0.1164). Overall, we note the possibility that future studies with a larger sample size may show that vaccination responses may improve as the individuals undergo dewilding, although our current analysis do not show statistical significance.

## Discussion

Understanding the effects of environmental transitions on the immune system could provide significant insights into the basis of immune variation between individuals and lead to novel therapeutics aimed to improve immunological diseases. Rewilding laboratory mice into natural outdoor enclosures has demonstrated distinct alterations to immune phenotypes from environmental change, including expanded granulopoiesis and circulation of peripheral blood neutrophils, bringing laboratory mice closer to human-like immune characteristics^13,16^. Moreover, creating inbred mice that have microbiomes of wild mice leads to enhanced survival after viral infection^35^. Hence, environmental factors can have significant influence on immunity. Here, we have studied the reverse transition, employing a “dewilding” model, where we transitioned rhesus macaques from a provisioned outdoor setting to a controlled indoor environment. In direct contrast to the rewilding studies, we found a significant reduction in neutrophils and an expansion of lymphocytes in the peripheral blood following dewilding.

An important limitation of our study is that we lacked age-matched wild controls in our analysis because this is logistically impossible. While we found that reduction of neutrophils and expansion of lymphocytes did not occur in age-matched African green monkeys (AGMs) that were bred in captivity and had not undergone environmental changes, AGMs differ substantially from rhesus macaques in T cell development. A more appropriate control would have been either longitudinal analysis of rhesus macaques that were age matched, born in captivity, and had not undergone environmental changes, or longitudinal analysis of rhesus macaques that were maintained in the provisioned outdoor environment. However, it is not feasible to obtain such samples. That AGM bred in captivity did not manifest reduced neutrophils during a similar age window is consistent with our observations in rhesus being attributed to the dewilding. While the AGM are natural hosts for SIVagm and their CD4 T cells down-regulate CD4 with peripheral immunological experience, these do not occur during early life^31^. Moreover, we’ve not seen hematological differences between lymphocyte, monocyte, and granulocyte counts between young AGM and RM. Thus, we believe longitudinal analysis of AGM born in captivity are a reasonable control for our “dewilded” rhesus macaques.

In humans, intestinal fungal colonization by *C. albicans* is associated with increased differentiation of T_H_17 CD4^+^ T cells^36^, and our previous studies on the microbiota of rewilded mice showed a significant increase in fungal colonization^13,16^. Thus, in contrast to rewilding, dewilding induces a shift from Type 17 response toward a Type 1 response and a decreased fungi-to-bacteria ratio in fecal contents. However, we also found an increase in central and effector T cells, alongside a decrease in naïve T cell frequencies during dewilding, which mirrors the same trajectory from rewilding studies^16^. These observations indicate that both models of environmental change stimulate maturation of the T cell compartment, likely due to altered antigenic exposure and immune system activation. One limitation of our analyses is that we did not measure circulating levels of cytokines and chemokines in the blood during dewilding, which could have supported our hypothesis about the shift from a Type 17 to a Type 1 response during dewilding.

In this study we had the rare opportunity to characterize changes to the hemopoietic cells in the BM during a major environmental change. By flow cytometry and scRNA-seq, we found significant changes to granulocyte and lymphocyte populations during dewilding. However, the changes observed in the peripheral blood during dewilding were not mirrored in the BM. In peripheral blood, dewilding led to reduced neutrophils and increased lymphocytes, whereas in the BM, we found increased granulocytes and reduced lymphocytes. An important limitation of this observation is that freezing and thawing of bone marrow cells could more severely affect granulocyte populations than other cell types. Alternatively, during dewilding we observed a shift towards a proinflammatory T_H_1 response, producing proinflammatory cytokines TNF-α and IFN-γ, which could promote lymphocyte egress from the BM, allowing granulocyte precursors to expand into the vacated space and undergo maturation. This dynamic would be consistent with our observations of increased expression of neutrophil maturation markers, such as CD11b and CD10^37,38^, in BM granulocytes, alongside accumulating CD87^hi^ mature neutrophils^39^ during dewilding.

During dewilding, the most notable change to hematopoietic stem and progenitor cell (HSPC) populations was a reduction in the overall HSPC compartment, identified by CD34^+^ cells, and in the more committed progenitors, defined as CD38^+^ cells. We found a shift from more differentiated progenitors, such as the common myeloid progenitors (CMP), towards the less committed multipotent progenitor (MPP) cells. These changes align with previously described regulation of hematopoietic stem cells (HSCs) during inflammation^40–42^, particularly influenced by TNF-α, which was elevated in our study. Although TNF-α was traditionally understood to inhibit growth and induce apoptosis in HSCs^43,44^, recent studies have shown that TNF-α promotes survival of more primitive HSC subpopulations while inducing apoptosis in more committed progenitors^45,46^. This may lead to an increased proportion of MPP cells in the BM, consistent with our findings during dewilding.

We also observed an increase in erythroid cells and progenitors in the BM, although this increase was not reflected in peripheral blood red blood cell counts, hematocrit, or hemoglobin levels during dewilding. The expression of genes related to hemoglobin control, including HBA, HBE1, and HBM, were significantly increased during dewilding, and their expression was localized to the expanded erythroid population in the BM during dewilding. Erythropoiesis can induce the egress of lymphocytes from the BM to the periphery and may reduce HSCs in the BM^47,48^. One hypothesis is that there is increased interplay between inflammation and erythropoiesis during dewilding, particularly mediated by TNF-α. Inflammation can trigger stress erythropoiesis that compensates for reduced steady-state erythropoiesis^49,50^, while erythropoietin can then resolve inflammation by suppressing NFκB signaling^51^. Perhaps this inflammation–erythropoiesis axis^52^ may contribute to the shifts we observed in BM composition during dewilding.

Because vaccines are frequently less effective in populations from developing countries, where microbial and antigen exposure is increased compared to more industrialized areas^53,54^, we sought to investigate if dewilding influences vaccine responsiveness. Although our results were not statistically significant, there was a trend suggesting that earlier vaccination relative to the onset of dewilding was associated with lower response to measles vaccination. In contrast, macaques vaccinated later in the dewilding process may respond better to the measles vaccine and exhibited higher antibody levels post-vaccination. Future studies with a larger sample size are needed to further test if vaccination responses could improve as the individuals undergo dewilding.

Another consideration is the appreciable stress the animals undergo during the transition from the outdoor-provisioned environment to the indoor environment. The social hierarchy is altered, the animals are exposed to more human interactions, and begin living in enclosures, causing significant stress that can also influence immunity. Further research is required to better understand the relationship between immune phenotypes and human social activity, especially since contact between individuals has significantly changed since the COVID-19 pandemic. It is also possible that biological sex may contribute to immunological alterations occurring during environmental changes. Unfortunately, female NHPs are generally rarely used for biomedical research to maintain colony sizes.

One possibility for the heightened T_H_1 response observed during dewilding may be infection with rhesus cytomegalovirus (RhCMV), common in captive rhesus macaques^55^. Indeed, all animals within our indoor environment are RhCMV seropositive, but we do not know if the macaques acquire CMV in the process of dewilding. Like humans, rhesus macaques typically exhibit overt CMV clinical symptoms only under immunosuppressive conditions^56,57^, but CMV infection drives large T cell clonal expansions^58,59^. It is also difficult to disentangle the individual components of the dewilding environment– such as altered diet, spatial confinement, and changes in antigen exposure– and their effects on the immune system. Nevertheless, this complexity mirrors the multifaceted nature of human migration from rural developing communities to more industrialized urban environments, where multiple environmental changes also occur simultaneously.

In summary, our results indicate that dewilding in rhesus macaques reverses some of the hematological effects of rewilding, with reduced peripheral blood neutrophils and increased peripheral lymphocytes. However, dewilding continues to mature the T cell compartment, increasing the proportion of memory T cells and reducing naïve T cells, coinciding with a shift to a T_H_1 response from a more T_H_17 response. We also documented changes to the BM during dewilding, including increased granulopoiesis, and changes in hematopoietic stem and progenitor cell populations. These shifts were accompanied by increased erythropoiesis, suggesting a dynamic interplay between immunological changes and erythropoiesis during dewilding. Overall, this study characterized some of the profound impacts of environmental change on the immune system. By describing the immunological consequences of transitioning to a more sterile environment in this rhesus macaque dewilding model, future studies may eventually inform important considerations for optimizing immune interventions, such as vaccination, in human migrants undergoing environmental transition.

## Methods

### Study design

The aims of this study were to examine how the environmental change of dewilding, the transition from a provisioned outdoor environment to indoor research facilities, affects the immune system of rhesus macaques. In study 1, 16 rhesus macaques (all male, with an average age of 2 years), were assessed before and after a 3-month quarantine in a lab environment. The macaques were cohoused in pairs to simulate social living conditions, and their diet was standardized to a controlled laboratory diet, which was consistent throughout the study period. Measurements included complete blood count (CBC) with differential of whole blood, in-vitro stimulation of whole blood and cytokine profiling with the LEGENDplex assay (BioLegend), and peripheral blood mononuclear cells (PBMC) isolation for spectral flow cytometry.

In study 2, 22 rhesus macaques (all male, with an average age of 1 year) were assessed at weeks 1, 3, 5, 7, and 9 of the dewilding quarantine period. Similarly to study 1, the macaques were cohoused in pairs, and their diet was maintained consistently with the laboratory standard. Measurements included CBC with differential of whole blood, spectral cytometry of BM, and single-nucleus RNA-sequencing of BM samples. We also evaluated the measles vaccination response in macaques during the dewilding process by randomly assigning 11 macaques into two groups: early vaccination at week 9 or late vaccination at week 23 of dewilding. Serum samples were collected 5 weeks following vaccination to assess the immune response.

For our analysis of the fungal and bacterial composition of fecal samples during dewilding, we assessed a third group of 17 dewilded rhesus macaques (all male). These macaques were housed consistently with the conditions of the other studies, including cohousing in pairs and maintaining a standardized laboratory diet. Fecal samples were collected at day 7 and day 18 of the dewilding process, and DNA was then extracted from the stool samples to analyze fungal and bacterial composition with qPCR.

For all analyses, samples that failed quality control, such as flow cytometry staining errors, were not included in downstream statistical analyses. The number of animals per group included in statistical analyses and the statistical tests used are reported in the figure legends. All data points are biological replicates.

### Animals

We collected data from 55 rhesus macaques for analyses. All rhesus macaques were male and aged at either 1 or 2 years. These rhesus macaques were given one dose of ivermectin (0.2 mg/kg of body weight by subcutaneous injection) following their initial exam as well as fenbendazole (50 mg/kg by oral administration) once a day for 3 days after the initial exam. Samples were taken 1 week after the initial exam (Week 1) and then throughout the dewilding quarantine period, as previously described.

The NIAID institutional animal care and use committee, as part of the NIH intramural research program, approved all experimental procedures pertaining to NHPs (protocol LVD 26E). The animals in this study were housed and cared for under the supervision of the Association for the Assessment and Accreditation of Laboratory Animal Care (AAALAC)-accredited Division of Veterinary Resources and as recommended by the Office of Animal Care and Use nonhuman primate management plan. Care at these facilities met the standards set forth by the Animal Welfare Act, animal welfare regulations, United States Fish and Wildlife Services regulations, as well as the 8th edition of the Guide for the Care and Use of Laboratory Animals^60^. The physical conditions of the animals were monitored daily. The animals were provided continuous access to water and offered commercial monkey biscuits twice daily as well as fresh produce, eggs and bread products, and a foraging mix consisting of raisins, nuts and rice. Enrichment to stimulate foraging and play activity was provided in the form of food puzzles, toys, cage furniture, and mirrors. All animals had the same base diet (LabDiet, St. Louis, MO, USA).

### Sample collection and CBC

Whole blood samples (approximately 30–50 μl) were collected at the timepoint of interest into EDTA-containing tubes for both spectral cytometry and complete blood count with differential (CBC). CBC was completed using the Element HT5 Veterinary Hematology Analyzer (Heska). Plasma was isolated from whole blood by centrifugation at 2000 rpm for 10 min at 22 °C. BM blood samples were aspirated from long bones into EDTA-containing tubes. For BM sample collection, rhesus macaques were sedated using tiletamine and zolazepam (Telazol) at a dosage of 3 to 4 mg/kg administered intramuscularly. Additionally, Buprenorphine Extended-Release, a compounded opioid providing up to 48 h of analgesia, was administered subcutaneously to ensure adequate pain management during and after the procedure.

Stool was collected from the bottom of individual macaques’ cages, placed inside polypropylene tubes, then flash frozen on dry ice before being stored at −80 °C.

### Preparation of single-cell suspensions for PBMCs and BM

Heparinized whole blood was spun for 10 min at 1500 rpm and plasma was collected and stored at −80 °C for further cytokine analysis. The cellular component re-suspended in PBS next underwent a density gradient separation process using the Lymphocyte Separation Media (LSM MP Biomedicals) according to the manufacturer’s instructions. Isolated PBMCs were washed twice in PBS and then used for downstream spectral cytometric analysis. PBMC isolation was performed on samples from the 16 macaques from study 1.

Single-cell suspensions were prepared from BM blood samples by thawing with water bath and warm RPMI, 2 washes with RPMI followed by centrifuging at 300 × *g* for 7 min and lysing red blood cells in 1 mL ACK Lysis buffer (Thermo Fisher Scientific) for 2 min.

### Spectral cytometry

Single-cell suspensions prepared from PBMCs or BM were washed twice with phosphate buffered saline (PBS) before incubating with Live/DeadFixable Blue (Thermo Fisher Scientific) for 20 min at 4 °C. For the intracellular PBMC panel, cells were stimulated with PMA and Ionomycin for 4 h and then incubated in eBioscience Transcription Factor Fixation and Permeabilization solution (Invitrogen) for 12–18 h at 4 °C and stained with cocktails of fluorescently labeled antibodies against intracellular antigens (full antibody list is in Fig. S3) and Fc Block (Human TruStain, BioLegend) diluted in Permeabilization Buffer (Invitrogen). For the remaining surface receptor panels, cocktails of fluorescently conjugated antibodies (full antibody list is in Figs. S3 and S6) diluted in FACS Buffer (0.5 g BSA, 1 mL EDTA, 500 mL PBS), Fc Block (Human TruStain, BioLegend), and 10% brilliant stain buffer (BD) were then added directly to cells and incubated for a further 30 min at 4 °C. Spectral unmixing was performed for each experiment using single-strained controls using UltraComp eBeads (Invitrogen). Dead cells and doublets were excluded from analysis. All samples were collected on an Aurora spectral cytometer (Cytek) and analyzed using the OMIQ platform (https://omiq.ai/) for manual gating and clustering of different populations^34^. Gating strategies are shown in Figs. S4, S8–11.

### In vitro stimulation

A single-cell suspension of PBMCs was reconstituted in RPMI at 2 × 106 cells per milliliter, and 0.1 ml was cultured in 96-well microtiter plates that contained 10^7^ colony-forming units per milliliter of UV-killed microbes or PBS control. The stimulated microbes are as following: *B. vulgatus* (ATCC 8482), *C. albicans* (UC820), *C. perfringens* (NCTC 10240), *S. aureus* (USA300 AH-LAC), *P. aeruginosa* (PAO1), *B. subtilis* (ATCC 6633), and House Dust Mite (HDM; Stallergenes Greer) prepared in house as previously published^33,61^. Supernatants were collected after 2 days and stored at −80 °C. Concentrations of IL-23, IL-12p40, CXCL10, IL-6, IL-10, IL-8, IL-1β, IFN-γ, CCL2, IFN-β, TNF-α, GM-CSF, and IL-17 in supernatants were measured using a commercially available human T helper cytokine LEGENDplex assay (Biolegend) according to the manufacturer’s instructions. Cytokine levels that were lower than the limit of detection across samples were excluded from further analysis.

### Single-nuclear RNA-seq library construction

Using the Chromium Next GEM Single Cell 3’ Kit v3.1, 4 reactions (1000269, 10X Genomics), Chromium Next GEM Single Cell 3ʹ Gel Bead Kit v3.1, 16 reactions (PN-1000122) and Chromium Next GEM Chip G Single Cell Kit (PN-1000120, 10X Genomics), nuclei isolated from 4 frozen BM samples (10X Protocol: Isolation of Nuclei for Single Cell RNA Sequencing & Tissues for Single Cell RNA Sequencing) were processed to generate single-cell Gel Beads-in-emulsion (GEMs) according to the manufacturer’s protocol. Approximately 2000 nuclei per sample were loaded onto a Chromium Single Cell Controller to create GEMs. Cells were lysed, and poly(A) RNA was barcoded during reverse transcription using a Thermo Fisher Veriti 96-well thermal cycler at 53 °C for 45 min, followed by 85 °C for 5 min. The resulting cDNA was generated and amplified. Quality control and quantification of the cDNA were performed using the Agilent Genomic DNA ScreenTape Analysis kit (5067-5366 for Genomic DNA Reagents and 5067-5365 for Genomic DNA ScreenTape) in the TapeStation system. Single-cell RNA-seq libraries were constructed using the Chromium Next GEM Single Cell 3ʹ Library Kit v3.1 (PN-1000158, 10X Genomics) and sequenced on a NovaSeq Xplus platform. The sequencing run was configured as a 28:10:10:90 cycle run, targeting 10,000 cells with 25,000 reads per cell.

### Analysis of single-nuclear RNA-seq data

The analysis single-nucleus RNA-seq samples was performed with the Cell Ranger 9.0.1 software. After assessing the UMI counts vs. barcodes graphs, the number of cells captured ranged from 1250 to 1400 and the mean reads per cell ranged from 84,790 to 214,143. Cells with low numbers of UMI counts were filtered out. The results from Cell Ranger were processed in R 4.5.0 with the Seurat (v5.3.0) package. Quality control filtering was applied to each sample to eliminate downstream analysis of empty droplets, low-quality cells, and potential doublets. Then, we used the Seurat package to perform calculation of the number of unique genes detected in each cell (nFeature_RNA), the total number of molecules detected within a cell (nCount_RNA), and the percentage of reads that map to the mitochondrial genome. We selected the 900 cells from each sample with the highest nFeature_RNA that also contained <10% mitochondrial reads, which limited sample 3–706 cells, leading us to take a random sample of 706 cells from the other 3 samples. Data were normalized and scaled prior to dimensionality reduction using principal component analysis (PCA). An elbow plot was used to determine the number of principal components for downstream analysis. Unsupervised clustering was performed using a shared nearest neighbor modularity optimization algorithm, followed by non-linear dimensionality reduction with UMAP for visualization. Marker genes for each cluster were identified using Wilcoxon rank-sum tests and then utilized to annotate clusters manually. Differentially expressed genes (DEGs) between dewilding timepoints were computed using the FindMarkers function with multiple thresholds. Visualization included PCA and UMAP plots, violin plots, dot plots, and heatmaps of top marker genes. All figures and tables were generated in R (v4.5.0).

### Determination of fungal and bacterial quantity during dewilding by qPCR

DNA was isolated from fecal samples with bead beating followed by Qiagen Symphony DNA extraction, as previously described^62^. We then performed quantitative PCR for fungus with the FungiQuant assay and for bacteria with a 16S assay. We used the TaqMan™ Fast Advanced Master Mix for qPCR (Thermo Fisher Scientific cat. 4444963). We tested 2 μL of DNA at a concentration of 0.1 ng/µL. The sequence of the forward primer for FungiQuant was 5′-GGRAAACTCACCAGGTCCAG-3′, the reverse primer sequence was 5′-GSWCTATCCCCAKCACGA-3′, and the probe sequence was 5′-TGGTGCATGGCCGTT-3′. For the 16S assay, the sequence of the forward primer was 5’- CGGTGAATACGTTCYCGG -3′, the reverse primer sequence was 5′-GGWTACCTTGTTACGACTT-3′, and the probe sequence was 5′-CTTGTACACACCGCCCGTC-3′. The standard curve for each PCR run was generated using FRODO 16S standards^32^ to calculate the bacterial copy number. Reaction conditions were the following: 50 °C for 2 min, 95 °C for 2 min, and 45 cycles of 95 °C for 1 s, followed by 60 °C for 20 s. We used the QuantStudio™ 3 System PCR machine (Thermo Fisher Scientific), and we performed the analysis with QuantStudio™ Design and Analysis Software (Thermo Fisher Scientific).

### Determination of measles titers by ELISA

Measles titers from serum samples were quantified using a commercially available Human Measles Virus IgG (MV IgG) enzyme-linked immunosorbent assay (ELISA) kit (Abbexa). Samples were assayed at a 101-fold dilution per the manufacturer’s protocol. Relative Measles IgG concentrations pre and post-vaccination were calculated based on optical density readings.

### Statistical analyses

Statistical analyses were conducted using Prism version 10 (GraphPad Software Inc.) Statistical parameters including the exact number (N) of rhesus macaques are annotated in the corresponding figure legend. Data presented in bar plots display the mean. Assumptions of normality and homogeneous variance were assessed to guide selection of statistical tests. Differences between dewilding timepoints were assessed using paired two-tailed *t* tests, unless otherwise indicated. Numerical *P* values are indicated in the figures, with *P* values ≤ 0.05 considered significant.

### Visualization

Data were visualized using R studio (v4.5.0), GraphPad Prism software (v10.5.0), and Seurat (Seurat v5.3.0; scRNA-seq data). Cartoon graphics were created using BioRender.com.

### Reporting summary

Further information on research design is available in the Nature Portfolio Reporting Summary linked to this article.

## Supplementary information

Supplementary Information

Reporting Summary

Transparent Peer Review file

## Source data

Source Data

## Data Availability

RNA-seq data were deposited into the Gene Expression Omnibus database: https://www.ncbi.nlm.nih.gov/geo/query/acc.cgi?acc=GSE301748. Source data are provided with this paper.
