# Supplementary material for: Hematological consequences of environmental change during dewilding of rhesus macaques
Source: Nat Commun. 2026 Jul 10;17:8522. doi: 10.1038/s41467-026-75260-w (PMC13483864; doi:10.1038/s41467-026-75260-w)
Supplement: Supplementary file 2 [file 41467_2026_75260_MOESM2_ESM.pdf]

nature portfolio

Corresponding author(s): Png Loke and Jason Brenchley

Last updated by author(s): Oct 15, 2025

Reporting Summary

Nature Portfolio wishes to improve the reproducibility of the work that we publish. This form provides structure for consistency and transparency in reporting. For further information on Nature Portfolio policies, see our Editorial Policies and the Editorial Policy Checklist.

Statistics

For all statistical analyses, confirm that the following items are present in the figure legend, table legend, main text, or Methods section.

n/a	Confirmed	
[ ]	[x]	The exact sample size (*n*) for each experimental group/condition, given as a discrete number and unit of measurement
[ ]	[x]	A statement on whether measurements were taken from distinct samples or whether the same sample was measured repeatedly
[ ]	[x]	The statistical test(s) used AND whether they are one- or two-sided<br>*Only common tests should be described solely by name; describe more complex techniques in the Methods section.*
[ ]	[x]	A description of all covariates tested
[ ]	[x]	A description of any assumptions or corrections, such as tests of normality and adjustment for multiple comparisons
[ ]	[x]	A full description of the statistical parameters including central tendency (e.g. means) or other basic estimates (e.g. regression coefficient) AND variation (e.g. standard deviation) or associated estimates of uncertainty (e.g. confidence intervals)
[x]	[ ]	For null hypothesis testing, the test statistic (e.g. *F*, *t*, *r*) with confidence intervals, effect sizes, degrees of freedom and *P* value noted<br>*Give P values as exact values whenever suitable.*
[x]	[ ]	For Bayesian analysis, information on the choice of priors and Markov chain Monte Carlo settings
[x]	[ ]	For hierarchical and complex designs, identification of the appropriate level for tests and full reporting of outcomes
[x]	[ ]	Estimates of effect sizes (e.g. Cohen's *d*, Pearson's *r*), indicating how they were calculated

Our web collection on statistics for biologists contains articles on many of the points above.

Software and code

Policy information about availability of computer code

Data collection	All the data collected in this study is primary data and not extracted from databases.
Data analysis	Statistical analyses were conducted using Prism version 10 (GraphPad Software Inc.). Data were visualized using R studio (v4.5.0), GraphPad Prism software (v10.5.0), and Seurat (Seurat v5.3.0; scRNA-seq data). Cartoon graphics were created using BioRender.com.

For manuscripts utilizing custom algorithms or software that are central to the research but not yet described in published literature, software must be made available to editors and reviewers. We strongly encourage code deposition in a community repository (e.g. GitHub). See the Nature Portfolio guidelines for submitting code & software for further information.

Data

Policy information about availability of data

All manuscripts must include a data availability statement. This statement should provide the following information, where applicable:

- Accession codes, unique identifiers, or web links for publicly available datasets
- A description of any restrictions on data availability
- For clinical datasets or third party data, please ensure that the statement adheres to our policy

RNA-seq data were deposited into the Gene Expression Omnibus database under accession number GSE301748. There are no restrictions on data availability.

Research involving human participants, their data, or biological material

Policy information about studies with human participants or human data. See also policy information about sex, gender (identity/presentation), and sexual orientation and race, ethnicity and racism.

Reporting on sex and gender	n/a
Reporting on race, ethnicity, or other socially relevant groupings	n/a
Population characteristics	n/a
Recruitment	n/a
Ethics oversight	n/a

Note that full information on the approval of the study protocol must also be provided in the manuscript.

Field-specific reporting

Please select the one below that is the best fit for your research. If you are not sure, read the appropriate sections before making your selection.

☒ Life sciences ☐ Behavioural & social sciences ☐ Ecological, evolutionary & environmental sciences

For a reference copy of the document with all sections, see nature.com/documents/nr-reporting-summary-flat.pdf

Life sciences study design

All studies must disclose on these points even when the disclosure is negative.

Sample size	No sample size calculation was formally performed. All available animals were used in the study.
Data exclusions	No data was excluded from the analysis.
Replication	The study describes two separate dewilding studies, for which some observations were replicated and others were not. This is stated in the results section.
Randomization	There was no randomization.
Blinding	There was no blinding.

Reporting for specific materials, systems and methods

We require information from authors about some types of materials, experimental systems and methods used in many studies. Here, indicate whether each material, system or method listed is relevant to your study. If you are not sure if a list item applies to your research, read the appropriate section before selecting a response.

Materials & experimental systems

n/a	Involved in the study	
☐	☒	Antibodies
☒	☐	Eukaryotic cell lines
☒	☐	Palaeontology and archaeology
☐	☒	Animals and other organisms
☒	☐	Clinical data
☒	☐	Dual use research of concern
☒	☐	Plants

Methods

n/a	Involved in the study	
☒	☐	ChIP-seq
☐	☒	Flow cytometry
☒	☐	MRI-based neuroimaging

Antibodies

Antibodies used	Antibodies used in flow cytometry panels are described in Supplementary Figure S3 and S6.

Validation	All flow cytometry antibodies were obtained from commercial sources with prior validation by the manufacturer. This was confirmed in house by titration assays and FMO analysis.

Animals and other research organisms

Policy information about studies involving animals; ARRIVE guidelines recommended for reporting animal research, and Sex and Gender in Research

Laboratory animals	Macaques (Macaca mulatta) utilized in this study were outbred and aged 1-2 years.
Wild animals	No animals were wild.
Reporting on sex	Female macaques are needed to maintain the colony. All study animals were male. Findings are likely applicable across sexes, but results may only pertain to male.
Field-collected samples	No samples were collected in the field.
Ethics oversight	The NIAID Division of Intramural Research Animal Care and Use Program, as part of the NIH Intramural Research Program, approved all the experimental procedures (animal study protocol LVD26E). The Program complies with all applicable provisions of the Animal Welfare Act and other federal statutes and regulations relating to animals. Animals were housed and cared for at the NIH Animal Center, under the supervision of the Association for the Assessment and Accreditation of Laboratory Animal Care (AAALAC)-accredited Division of Veterinary Resources and as recommended by the Office of Animal Care and Use Nonhuman Primate Management Plan. Husbandry and care met the standards set forth by the Animal Welfare Act, Animal Welfare Regulations, as well as The Guide for the Care and Use of Laboratory Animals (8th Edition). The physical conditions of the animals were monitored daily. Animals were provided continuous access to water and offered commercial monkey biscuits twice daily as well as fresh produce, eggs, and bread products twice weekly and a foraging mix consisting of raisins, nuts, and rice thrice weekly. Enrichment to stimulate foraging and play activity was provided in the form of food puzzles, toys, cage furniture, and mirrors or television. All procedures were carried out under ketamine anesthesia by trained personnel under the supervision of veterinary staff, and all efforts were made to maximize animal welfare and to minimize animal suffering in accordance with the recommendations of the Weatherall report on the use of NHPs. Animals were housed in adjoining individual primate cages, allowing social interactions, under controlled conditions of humidity, temperature, and light (12-hour light/12-hour dark cycles). Experimental endpoints were determined by veterinarian staff based upon clinical findings. No animal experienced overt clinical symptomology.

Note that full information on the approval of the study protocol must also be provided in the manuscript.

Plants

Seed stocks	n/a
Novel plant genotypes	n/a
Authentication	n/a

Flow Cytometry

Plots

Confirm that:

☒ The axis labels state the marker and fluorochrome used (e.g. CD4-FITC).

☒ The axis scales are clearly visible. Include numbers along axes only for bottom left plot of group (a 'group' is an analysis of identical markers).

☒ All plots are contour plots with outliers or pseudocolor plots.

☒ A numerical value for number of cells or percentage (with statistics) is provided.

Methodology

Sample preparation	Whole blood samples were collected at the timepoint of interest into EDTA-containing tubes. Bone marrow samples were aspirated from long bones into EDTA-containing tubes. Heparinized whole blood was spun for 10min at 1,500rpm and plasma was collected and stored at −80°C for further cytokine analysis. The cellular component re-suspended in PBS next underwent a density gradient separation process using the Lymphocyte Separation Media (LSM MP Biomedicals) according to the manufacturer's instructions. Isolated PBMCs were washed twice in PBS and then used for downstream spectral cytometric analysis. PBMC isolation was performed on samples from the 16 macaques from study 1.

	Single-cell suspensions were prepared from BM blood samples by thawing with water bath and warm RPMI, 2 washes with RPMI followed by centrifuging at 300 g for 7 min and lysing red blood cells in 1 mL ACK Lysis buffer (Thermo Fisher Scientific) for 2 minutes. Single-cell suspensions prepared from PBMCs or BM were washed twice with phosphate buffered saline (PBS) before incubating with Live/DeadFixable Blue (Thermo Fisher Scientific) for 20 min at 4ºC. For the intracellular PBMC panel, cells were next incubated in eBioscience Transcription Factor Fixation and Permeabilization solution (Invitrogen) for 12–18 h at 4°C and stained with cocktails of fluorescently labeled antibodies against intracellular antigens (full antibody list is in Figure S3) and Fc Block (Human TruStain, BioLegend) diluted in Permeabilization Buffer (Invitrogen). For the remaining surface receptor panels, cocktails of fluorescently conjugated antibodies (full antibody list is in Figures S3 and S6) diluted in FACS Buffer (0.5g BSA, 1 mL EDTA, 500 mL PBS), Fc Block (Human TruStain, BioLegend), and 10% brilliant stain buffer (BD) were then added directly to cells and incubated for a further 30 min at 4ºC. Spectral unmixing was performed for each experiment using single-strained controls using UltraComp eBeads (Invitrogen). Dead cells and doublets were excluded from analysis.
Instrument	Samples were collected on a Cytek Aurora Spectral Flow Cytometer
Software	Data was uploaded and analyzed on the cloud based system OMIQ.AI
Cell population abundance	n/a. No cell sorting.
Gating strategy	All the gating strategy is shown in Supplemental Figures

☒ Tick this box to confirm that a figure exemplifying the gating strategy is provided in the Supplementary Information.
