# Supplementary material for: Hematological consequences of environmental change during dewilding of rhesus macaques
Source: Nat Commun. 2026 Jul 10;17:8522. doi: 10.1038/s41467-026-75260-w (PMC13483864; doi:10.1038/s41467-026-75260-w)
Supplement: Supplementary file 3 [file 41467_2026_75260_MOESM3_ESM.pdf]

nature portfolio

Peer Review File

Hematological Consequences of Environmental Change During Dewilding of Rhesus Macaques

Corresponding Author: Dr Jason Brenchley

This file contains all reviewer reports in order by version, followed by all author rebuttals in order by version.

Version 0:

Reviewer comments:

Reviewer #1

(Remarks to the Author)
In this manuscript, Smith and colleagues explore how a transition from outdoor to indoor housing (termed "dewilding") affects peripheral immune composition, bone marrow hematopoiesis, and vaccine responsiveness in rhesus macaques. They report that dewilding increases circulating lymphocytes and decreases granulocyte frequencies. Using whole-blood stimulations and intracellular cytokine staining, the authors show a reduction in Th17 responses paralleled by an increase in Th1 responses, which they link to altered fungal and bacterial abundances in stool. Within the $CD4^+$ and $CD8^+$ compartments, dewilding decreases naïve and increases memory T-cell frequencies. Bone-marrow profiling reveals a shift toward less-committed multipotent progenitors (MPPs), increased $CD66^+$ granulocytes, and enhanced erythropoiesis. Finally, macaques vaccinated later in the dewilding process show a trend toward improved measles-vaccine responsiveness.

Overall, this is a unique and conceptually important study. It models immune adaptation during environmental transitions, analogous to human migration from rural to urban settings, and ambitiously integrates hematological, immunological, and transcriptomic data. The inclusion of bone-marrow analyses is particularly commendable. The discussion, however, is often speculative and should be toned down.

Here are a few specific comments and questions:

1.My main concern lies in the absence of an appropriate control for developmental maturation. Rhesus macaques reach sexual maturity between 2.5 and 3.5 years (PMCID: PMC5466843), whereas animals in Study 1 (~2 years) and Study 2 (~1 year) were still maturing. Many of the observed immunological shifts—decreased naïve T cells, increased memory T cells, altered hematopoietic composition—could reflect normal age-related development rather than environmental change per se. The authors attempt to address this by comparing African Green Monkeys (AGMs) that did not undergo dewilding (Fig. 1E), but the rationale for this choice is unclear. AGMs differ substantially from rhesus macaques in developmental timing and immune ontogeny. A more valid control would have been age-matched rhesus macaques kept in the outdoor environment over the same period. The manuscript should:

1A. Explicitly discuss this developmental confound and its likely magnitude.
1B. Justify the use of AGMs as controls or, if data exist, include cross-study comparisons between pre-quarantine (Study 1) and week 1 (Study 2) macaques to gauge baseline consistency.

Without addressing this issue, it remains difficult to disentangle maturation from environmental effects.

2. For Fig. 2A, considering the number of cytokines and stimuli being tested, is there any FDR adjustment being done?

3. In Figure 3, the gating for effector-memory (EM) subsets appears to use $CD45RA^+CCR7^-$ criteria, corresponding to EMRA rather than true EM cells (see PMC11463637). The authors should verify this gating and re-analyze if needed, as it affects key conclusions about memory differentiation.

4. Total fungal and bacterial qPCR lacks taxonomic resolution. Acknowledge this limitation clearly and avoid implying specific microbial causation.

5. The statistical handling of vaccination data requires revision. For Figure 7B, entering the responder/non-responder counts into a 2 × 2 table and performing Fisher's exact test yields P = 0.38, indicating no significant difference in responder proportions. For Figure 7C, direct comparison of optical-density values using a P-value is not appropriate. A more robust approach would be to perform a linear-regression model with an interaction term testing whether post-vaccination changes differ between early and late groups. These adjustments would provide a sounder statistical foundation for the conclusions.

Minor comments:
1. I suggest to report effect sizes alongside p-values

2. Fig S2 is quite overwhelming. I would advise to make a summarizing figure e.g. heatmap so that readers can quickly skim shifts in pathogen-specific cytokine responses.

3. Supplemental Figure 3: for panel B (intracellular markers) rows 10-18 were duplicate of rows 1-9

4. For figure 4, maybe add a stacked bar chart of cell frequencies to help readers quickly understand compositional changes between timepoints such as in Figure 6B

5. For figure 6A, report the total number of cells. Do the same for figure 6D.

6. I recommend to split schematics in figure 7A to two arms for early and late vaccinations instead of on one contiguous arrow. This could potentially mislead the reader.

Reviewer #2

(Remarks to the Author)
The authors addressed the impact of environmental change on microbiome and immune systems using a dewilding macaque model. While there are several limitations in this model to reflect actual situation in human population as authors described in the Discussion, study protocol and analyses have been well organized. It is important that some immunological changes observed in dewilding are coincidence to previous rewilding study whereas dewilding reverses some immunological change of rewilding. There are a few comments to improve the manuscript.

Minor points:
1. In Figure 2B, authors analyzed CD4+ Th1/Th17 response ratio by quantifying IFN-g and IL-17 production. Is it correct that these analyses were performed using PBMCs with non-specific stimulation such as PMA/Ionomycin? Authors should describe details of assay in Methods and Figure legend.
2. Authors discusses that inflammatory responses due to different pathogen exposure may contribute to the change of immune system. Do authors have any data about quantity of cytokine or chemokine in the blood before/after dewilding? These data may support the hypothesis authors described.
3. By COVID-19 pandemic, human social activities have been dramatically changed from the before. Especially, long-term reduced contact with each other during the pandemic resulted in increased or season-sifted epidemic of many infectious diseases thereafter. How do the results in this model reflect the change of human social activity?

Reviewer #3

(Remarks to the Author)
General
A. Smith et al. analyzed the effects of dewilding young rhesus macaques on hematological, immunological, and microbiological parameters. The subject is of interest because it has direct impacts on preclinical research and the welfare of animals, as well as from a translational perspective. Changes in environmental conditions lead to alterations in complete blood count values, such as a decrease in neutrophil count and an increase in lymphocyte count. Animals housed in laboratory settings exhibited an increase in CD4+ T central memory (T CM) and effector memory (T EM) cells, accompanied by a decrease in naïve CD4+ T cells. Overall, T cells are more activated, with a shift toward a Th1 response against antigens. This is associated with changes in fungal and bacterial microbiota as exposure to environmental factors is reduced in laboratory conditions. Finally, they analyzed the response to measles vaccination in relation to the duration of quarantine and found reduced responsiveness when animals were vaccinated early in the dewilding process. The manuscript is very clear and well written, with nice figures. The methodology is clear and documented. The scheme summarizing the results is also appreciated.

Major points
The project may have been conducted in the Primate Biomedical Facility and may have taken advantage of the ongoing quarantine for specific projects. So, I understand that the individuals chosen depend on the animals available and that some analyses are difficult to run or re-run.
Here are comments or questions that need to be addressed:
The study is based on the concepts of dewilding and rewilding, but it lacks a clear definition within the context of this research. To better understand environmental factors, it would be helpful to describe animal housing in "wild" conditions. For example, is it an outdoor enclosure where young animals (1 or 2 years old) live with adults in a larger group? Are the animals born in the facility, or have they been moved to outdoor enclosures after weaning and transportation from another breeding facility? The weaning age of animals could also have an impact on stress and immunological factors. This

suggests that the young rhesus macaques are not in contact with adults, which could have social/sanitary/microbiological impacts.
The second point is that the animals used in the study are still young macaques (1-2 years old), and studies have shown changes in hematological and immunological parameters during the first years of life. (doi: 10.3402/pba.v2i0.18052 , doi: 10.1007/s11357-023-00962-8 , https://doi.org/10.3389/fped.2020.00388)
In the current study, there is no "wild" control group, which could have been used to monitor these changes during quarantine. Therefore, it remains difficult to determine if the observed changes are partly related to animal aging. I also found it challenging to compare CBC values with AGM, as I have never experienced environmental changes, even though the dynamics might be similar; these two species originate from different geographical locations. Another limitation is that only male macaques were used, which may conceal other variations or dynamics present in female macaques.
The third point is that environmental factors are attributed to the immunological parameters, but Stress factors are not discussed. Dewilding is a significant sociological shift, as animals transition from a large group to a pair. Even in young animals, social status is changed and may also affect immune parameters (DOI: 10.1126/science.aah3580). Therefore, this point should be discussed, as the only change in social hierarchy has been shown to impact TLR4 signaling and pro-inflammatory responses.
Minor and detailed comments:
Line 245: As mentioned before, using AGM as wild controls is questionable but might be the only wild animals available in the facility. The comparison should be made with caution.
Line 248: Parentheses missing.
Line 249: Do you have an idea of the parasite/helminth circulation within "wild" animals in the facility? Variation of eosinophils is quite interesting, and your conclusion makes sense but is difficult to prove.
Line 250: The number of cytokines and stimulants used in this part is quite interesting and impressive, and could give a nice idea of the PBMC responses. How you selected each antigen would be of added value to better under the strategy used. Only stimulation with CA and BV showed differences in terms of cytokine PMC response. The reduction of IL-17 production with CA stimulation (and SA?), the increase of IL-12p40 secretion with BV, and the increase of CXCL10 (SA and CP), TNF-α (PA) support a shift toward a TH1 response after quarantine. I think citing also that CP, SA, and PA support your finding in this part would be a plus. These bacteria are not cited in the results part, whereas they reinforce the observations. Supplementary Figure 2 is very small; a bigger version would be appreciated.
Line 260: In the ICS experiment showing a reduction of IL17+ and an increase of IFNg in CD4+ T cells, it is not clear to me which antigen was used to stimulate the cells. Please specify which antigens you used, as you used several.
Line 268: Fecal samples were not taken by rectal swab but by feces collection in cages, if I understood well. This method may be associated with environmental contamination, which, in itself, differs between "wild" and laboratory conditions. Do you think this could impact your microbiota analysis?
Line 287: The bone marrow analysis seems to be performed on frozen cells. The freezing and thawing of blood and bone marrow can result in partial or complete depletion of granulocytes. The fact that results from BM do not reflect those from the blood might be due to a selective depletion of granulocytes. On supplementary figures 9 and 10, I'm surprised to observe so few FSC high SCC high granulocytes, whereas macaque bone marrow is composed of 70 to 80% granulocytes. Only the stem cells can be cryopreserved and analyzed here. I would analyze the phenotype of immature and mature neutrophils with caution, as the freezing process significantly alters these populations. Additionally, the classification used for CD11b and CD87 in neutrophils is unfamiliar to me. Could you cite the paper supporting this classification?
Line 345: The vaccine protocol used here seems to rely on only one shot of the measles vaccine. The vaccine used and the immunization protocol need to be described. The vaccine protocol in macaques typically involves two shots (administered 3 to 6 months apart) of MMR and is associated with seroconversion rates of 90-95%. So, if such a protocol has been used here, I'm surprised by the low level of seroconversion. Otherwise, if only one shot has been used, I'm not surprised, but this is not the standard protocol. The conclusion on these results should be cautiously revised in this regard.
Line 437: The Authors discussed the possibility that infection with RhCMV may influence the immune response observed in the study. This is a really good point, as these young animals are likely to get infected. Is it possible to show data on the seroconversion of macaques against CMV?

Version 1:

Reviewer comments:

Reviewer #1

(Remarks to the Author)
I have carefully reviewed the authors' point-by-point rebuttal and the accompanying revised figures. All major concerns have been substantively addressed. The developmental maturation confound is now openly discussed as a study limitation, with a fair justification for the AGM controls given the logistical constraints of the field site. The T cell memory gating has been corrected to properly distinguish T_EM from T_EMRA populations, the cytokine data now carry appropriate FDR correction, the microbiome conclusions have been tempered to reflect the limited taxonomic resolution of qPCR, and the vaccination results are presented as exploratory findings with full statistical transparency. Each minor point—effect sizes, figure clarity, data reporting, and schematic redesign—has also been resolved. The revised manuscript is more rigorous in its statistical framework, more measured in its interpretive claims, and more transparent about what the data can and cannot support.

I am satisfied that the authors have engaged constructively with all reviewer feedback and recommend acceptance of the revised manuscript.

Reviewer #2

(Remarks to the Author)
Authors have addressed all comments and modified the manuscript appropriately.

Reviewer #3

(Remarks to the Author)
Dear authors, the corrections and modifications made to the manuscript have answered my questions. The detailed information provided helped evaluate and support the study's conclusions.
I don't have any further comments.

Open Access This Peer Review File is licensed under a Creative Commons Attribution 4.0 International License, which permits use, sharing, adaptation, distribution and reproduction in any medium or format, as long as you give appropriate credit to the original author(s) and the source, provide a link to the Creative Commons license, and indicate if changes were made.
In cases where reviewers are anonymous, credit should be given to 'Anonymous Referee' and the source.
The images or other third party material in this Peer Review File are included in the article’s Creative Commons license, unless indicated otherwise in a credit line to the material. If material is not included in the article’s Creative Commons license and your intended use is not permitted by statutory regulation or exceeds the permitted use, you will need to obtain permission directly from the copyright holder.
To view a copy of this license, visit https://creativecommons.org/licenses/by/4.0/

We thank the reviewers for their careful assessment of our work. We are pleased that the reviewers and editorial board found our work to be of interest. We agree with all the important points raised by the Reviewers, and we have addressed all the points below. We have included more data, reanalyzed data and produced new visualizations. Most importantly, we acknowledge and discuss the caveat of lacking age matched wild controls in the Discussion.

The animals we "dewild" initiate life in a provisioned outdoor environment. This environment is only accessible by boat with very limited facilities. The only electricity comes from generators, used to provide electricity to trailers that house staff and there are no laboratory facilities. In this setting it would be impossible to perform the detailed immunological analyses we report in the manuscript. Moreover, it is impossible for detailed longitudinal biological samples to be obtained and processed, since the animals are free-ranging, with access to trees and many acres of open space. Hence, as important as this control group would be to analyze, it is beyond our capacity. We now carefully discuss these limitations in the revised manuscript.

REVIEWER COMMENTS

Reviewer #1 (Remarks to the Author):

In this manuscript, Smith and colleagues explore how a transition from outdoor to indoor housing (termed "dewilding") affects peripheral immune composition, bone marrow hematopoiesis, and vaccine responsiveness in rhesus macaques. They report that dewilding increases circulating lymphocytes and decreases granulocyte frequencies. Using whole-blood stimulations and intracellular cytokine staining, the authors show a reduction in Th17 responses paralleled by an increase in Th1 responses, which they link to altered fungal and bacterial abundances in stool. Within the $CD4^+$ and $CD8^+$ compartments, dewilding decreases naïve and increases memory T-cell frequencies. Bone-marrow profiling reveals a shift toward less-committed multipotent progenitors (MPPs), increased $CD66^+$ granulocytes, and enhanced erythropoiesis. Finally, macaques vaccinated later in the dewilding process show a trend toward improved measles-vaccine responsiveness.

Overall, this is a unique and conceptually important study. It models immune adaptation during environmental transitions, analogous to human migration from rural to urban settings, and ambitiously integrates hematological, immunological, and transcriptomic data. The inclusion of bone-marrow analyses is particularly commendable. The discussion, however, is often speculative and should be toned down.

We thank the Reviewer for their positive comments on the manuscript and their careful analysis of the work. We have addressed the points they've raised with a revised, and, we believe,

improved manuscript. This includes substantially rewriting and toning down the discussion as suggested. Details are included below in response to each point made.

Here are a few specific comments and questions:

1.My main concern lies in the absence of an appropriate control for developmental maturation. Rhesus macaques reach sexual maturity between 2.5 and 3.5 years (PMCID: PMC5466843), whereas animals in Study 1 (~2 years) and Study 2 (~1 year) were still maturing. Many of the observed immunological shifts—decreased naïve T cells, increased memory T cells, altered hematopoietic composition—could reflect normal age-related development rather than environmental change per se. The authors attempt to address this by comparing African Green Monkeys (AGMs) that did not undergo dewilding (Fig. 1E), but the rationale for this choice is unclear. AGMs differ substantially from rhesus macaques in developmental timing and immune ontogeny. A more valid control would have been age-matched rhesus macaques kept in the outdoor environment over the same period. The manuscript should:

1A. Explicitly discuss this developmental confound and its likely magnitude.

The Reviewer raises an important point but unfortunately, it is not possible to have age-matched outdoor macaques as controls. The animals we "dewild" initiate life in a provisioned outdoor environment. This environment is only accessible by boat with very limited facilities. The only electricity comes from generators, used to provide electricity to trailers that house staff and there are no laboratory facilities. In this setting it would be impossible to perform the detailed immunological analyses we report in the manuscript. Moreover, it is impossible for detailed longitudinal biological samples to be obtained and processed, since the animals are free-ranging, with access to trees and many acres of open space. Hence, as important as this control group would be to analyze, it is beyond our capacity. Additionally, we also cannot perform similar studies in rhesus that had not been "dewilded" because that requires indoor breeding, which is not feasible in the intramural program.

We carefully acknowledge this limitation in the discussion of our revised manuscript and thank the reviewer for highlighting this issue.

Line 451: "An important limitation of our study is that we lacked age-matched wild controls in our analysis because this is logistically impossible. While we found that reduction of neutrophils and expansion of lymphocytes did not occur in age-matched African green monkeys (AGMs) that were bred in captivity and had not undergone environmental changes, AGMs differ substantially from rhesus macaques in T cell development. A more appropriate control would have been either longitudinal analysis of rhesus macaques that were age matched, born in captivity, and had not undergone environmental changes, or longitudinal analysis of rhesus macaques that were maintained in the provisioned outdoor environment. However, it is not feasible to obtain such samples."

1B. Justify the use of AGMs as controls or, if data exist, include cross-study comparisons between pre-quarantine (Study 1) and week 1 (Study 2) macaques to gauge baseline consistency.

Without addressing this issue, it remains difficult to disentangle maturation from environmental effects.

We completely agree with the Reviewers that developmental maturation is an important caveat for this study. We tried our best to address this concern with data using the AGMs as controls (Figure 1E) and tracking CBC w/diff data on rhesus macaques in our colonies. However, these data do not address this concern as directly as data from age matched controls in the wild macaques.

We imported a small colony of vervet African green monkeys (AGMs) from Tanzania in the early 2000's. To maintain the colony of AGM, we have had some success in breeding the AGM. These animals are natural hosts for SIVagm and, as the reviewer correctly points out, have significantly different T cell development compared to rhesus. In particular, as animals experience immunological pressure, AGM CD4 T cells downregulate CD4 and upregulate the alpha chain (not beta) of CD8 (PMID 35750337 28438898 32841214 19525963). We believe this is a major immunological mechanism responsible for the nonprogressive nature of SIVagm infection in AGM. However, from our years of experience, this CD4 downregulation does not occur early in life and thymic development is "normal" (PMID 24991011).

Therefore, based on previous experiences, AGM and rhesus have similar immunological profiles early in life (similar CBC, blood chemistries, CD4/CD8 ratios, and frequencies of naïve/CM/EM T cells). We have no previous evidence to suggest hematopoiesis differs significantly between myeloid and lymphoid lineages between AGM and RM. Moreover, the comparisons we make are longitudinal, the differences we observe in rhesus during the "dewilding" process do not occur in AGM that are born in captivity. While we acknowledge that longitudinal analysis of RM born in captivity would have been a more appropriate control, we hope the reviewer appreciates the complexity of this study and that our AGM controls are reasonable.

In Figure 1, we compare Study 1 and Study 2 CBC data to show consistency of changes in neutrophils and lymphocytes from baseline.

We have now included the following explicit discussion of this developmental confounder and its potential magnitude in the Discussion section.

Line 458: "That AGM bred in captivity did not manifest reduced neutrophils during a similar age window is consistent with our observations in rhesus being attributed to the dewilding. While the AGM are natural hosts for SIVagm and their CD4 T cells down-regulate CD4 with peripheral immunological experience, these do not occur during early life[37]. Moreover, we've not seen hematological differences between lymphocyte, monocyte, and granulocyte counts between

young AGM and RM. Thus, we believe longitudinal analysis of AGM born in captivity are a reasonable control for our "dewilded" rhesus macaques."

2. For Fig. 2A, considering the number of cytokines and stimuli being tested, is there any FDR adjustment being done?

This is an excellent suggestion by the Reviewer, and we have now calculated FDR-adjusted p values and visualized the data as a heatmap in Figure S2 (see below), which also simplifies presentation of this dataset (as also suggested by the Reviewer under minor comments).

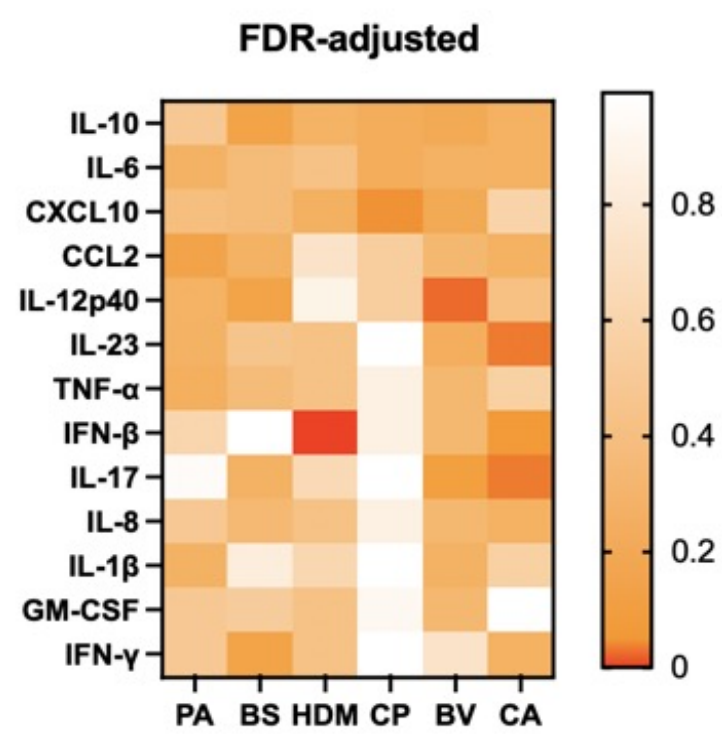

Figure S2. Dewilding-driven shifts in pathogen-specific immune responses. Heatmap of FDR-adjusted p-values of before vs after cytokine response to *in vitro* stimulation by *Pseudomonas aeruginosa* (PA), *Bacillus subtilis* (BS), House Dust Mite (HDM), *Clostridium perfringens* (CP), and *Bacteroides vulgatus* (BV), *Candida albicans* (CA), before and after quarantine (N=16).

3. In Figure 3, the gating for effector-memory (EM) subsets appears to use $CD45RA^+CCR7^-$ criteria, corresponding to EMRA rather than true EM cells (see PMC11463637). The authors should verify this gating and re-analyze if needed, as it affects key conclusions about memory differentiation.

We thank the Reviewer for this correction. We have now adopted the gating strategy suggested in PMC11463637 (added as a reference), reanalyzed the data and defined CD45RA+CCR7- cells as T_{EMRA} and the CD45RA-CCR7- cells as the T_{EM} cells. See below revised Figure 3.

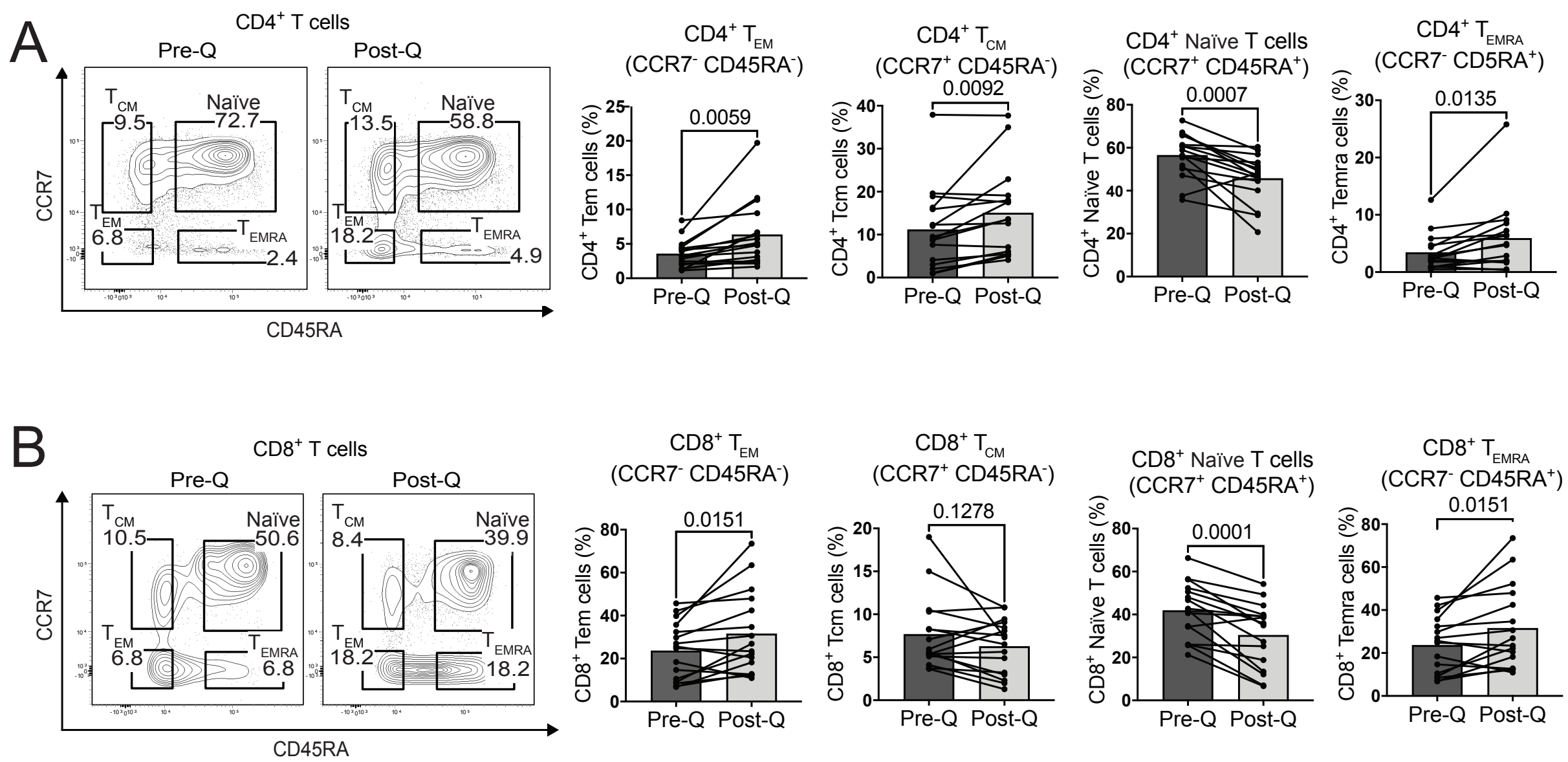

Figure 3. Dewilding-driven shifts in T-cell populations. (A-B) Representative flow cytometry analysis and quantification of Central Memory (T_{CM}), Effector Memory (T_{EM}), Effector Memory cells Re-expressing CD45RA (T_{EMRA}) and naïve T cells for $CD4^+$ (A) and $CD8^+$ (B) T cells, before and after quarantine (N=16). Points in bar plots represent individual rhesus macaques. *P* values were determined by paired two-tailed t-test.

4. Total fungal and bacterial qPCR lacks taxonomic resolution. Acknowledge this limitation clearly and avoid implying specific microbial causation.

The Reviewer makes a good point. We'd initially hoped to use either ITS sequencing or shotgun sequencing to explore the composition of the microbiome as the animal's transition. However, our ability to perform these experiments early in 2025 were influenced by significant budgetary and procurement constraints which were placed upon the intramural program and we were unable to acquire the data.

We added this to the Results section in Line 334: "However, these results lack taxonomic resolution without metagenomic sequencing of the microbiome, which was not feasible in this study."

We also removed this sentence from the Results section: "The decline in the relative fungal:bacterial ratio during dewilding might play a role in the shift from a Type 17 response to a Type 1 response."

5. The statistical handling of vaccination data requires revision. For Figure 7B, entering the responder/non-responder counts into a 2 × 2 table and performing Fisher's exact test yields P = 0.38, indicating no significant difference in responder proportions. For Figure 7C, direct

comparison of optical-density values using a P-value is not appropriate. A more robust approach would be to perform a linear-regression model with an interaction term testing whether post-vaccination changes differ between early and late groups. These adjustments would provide a sounder statistical foundation for the conclusions.

We thank the reviewer for this feedback regarding the statistical analysis of the vaccination data. We agree that there are not statistically significant differences between early and late groups and have significantly toned back our conclusions.

As the Reviewer suggested, we performed a linear regression model including an interaction term between time (pre- vs post-vaccination) and vaccination group (Early vs Late) to formally test whether post-vaccination changes differed between groups. In this model, the interaction term was not statistically significant (coefficient = 0.1853, $P = 0.309$). We also evaluated a simplified model in which the change in OD (ΔOD = post − pre) was regressed on the vaccination group alone ($R^2 = 0.052$, $P = 0.137$). Hence, the differences between Early vs Late cannot be considered statistically significant.

Because the vaccination cohort is small, both regression approaches are likely underpowered to detect the between-group differences. We have therefore revised the manuscript to (i) clarify that statistical analyses do not demonstrate a statistically significant difference between groups, likely resulting from being underpowered and (ii) frame the vaccination results as descriptive and exploratory.

Revised Results, Line 425: "We noted that among the early vaccination group, there was an approximately equal distribution of high responders (N = 5) and low responders (N = 6), whereas the late vaccination group showed a majority of macaques being high responders (N = 8) vs low responders (N = 3) (Figure 7B). However, these differences were not statistically significant by Fisher's exact test ($P = 0.38$) or by a linear regression model in which the change in OD was regressed on vaccination groups ($R^2 = 0.052$, $P = 0.137$), likely because the experiment is underpowered. Although the post-vaccination OD value was higher in the late vaccination group (0.5343) compared to the early vaccination group (0.3322), this difference was also not statistically significant ($p = 0.1164$). Overall, we note the possibility that future studies with a larger sample size may show that vaccination responses could improve as the individuals undergo dewilding, although our current analysis do not show statistical significance."

Minor comments:
1. I suggest to report effect sizes alongside p-values.

This has been added to the Figure Legends.

2. Fig S2 is quite overwhelming. I would advise to make a summarizing figure e.g. heatmap so that readers can quickly skim shifts in pathogen-specific cytokine responses.

This is an excellent suggestion from the Reviewer and we have now simplified Fig S2 as a heatmap of p-values as well as FDR adjusted p-values (see above)

3. Supplemental Figure 3: for panel B (intracellular markers) rows 10-18 were duplicate of rows 1-9

We thank the Reviewer for catching this error and it has been corrected.

4. For figure 4, maybe add a stacked bar chart of cell frequencies to help readers quickly understand compositional changes between timepoints such as in Figure 6B.

This is an excellent suggestion from the Reviewer. Please see revised Figure 6B.

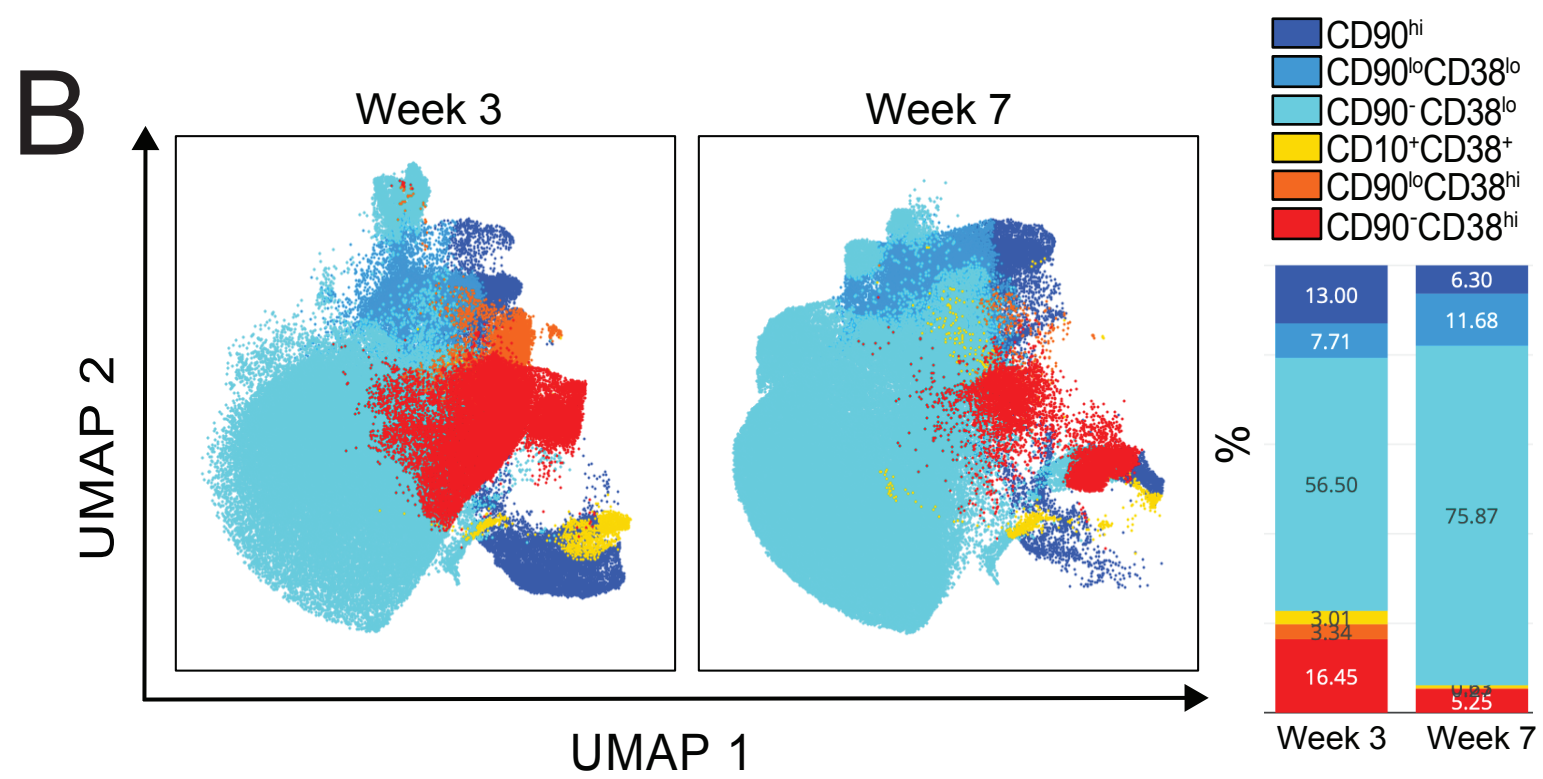

5. For figure 6A, report the total number of cells. Do the same for figure 6D.

The total number of cells is now reported in the Figure Legends.

6. I recommend to split schematics in figure 7A to two arms for early and late vaccinations instead of on one contiguous arrow. This could potentially mislead the reader.

This is an excellent suggestion and please see attached revised Figure 7A.

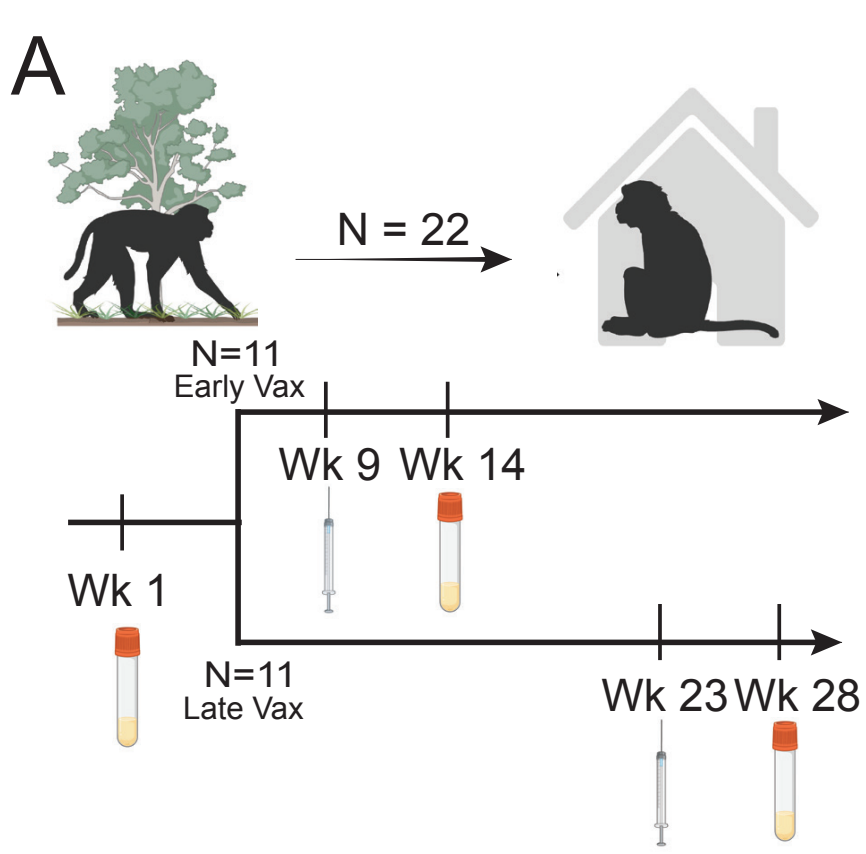

Reviewer #2 (Remarks to the Author):

The authors addressed the impact of environmental change on microbiome and immune systems using a dewilding macaque model. While there are several limitations in this model to reflect actual situation in human population as authors described in the Discussion, study protocol and analyses have been well organized. It is important that some immunological changes observed in dewilding are coincidence to previous rewilding study whereas dewilding reverses some immunological change of rewilding. There are a few comments to improve the manuscript.

Minor points:
1. In Figure 2B, authors analyzed CD4+ Th1/Th17 response ratio by quantifying IFN-g and IL-17 production. Is it correct that these analyses were performed using PBMCs with non-specific stimulation such as PMA/Ionomycin? Authors should describe details of assay in Methods and Figure legend.

The Reviewer is correct, and we apologize for the lack of details. This has now been updated in Materials and Methods, Results and Figure Legends.

2. Authors discusses that inflammatory responses due to different pathogen exposure may contribute to the change of immune system. Do authors have any data about quantity of cytokine or chemokine in the blood before/after dewilding? These data may support the hypothesis authors described.

Unfortunately, we did not measure cytokines and chemokines in circulation, and we no longer have sufficient plasma for these assays. We have added the following sentence to the Discussion.

Line 473: "One limitation of our analyses is that we did not measure circulating levels of cytokines and chemokines in the blood during dewilding, which could have supported our hypothesis about the shift from a Type 17 to a Type 1 response during dewilding."

3. By COVID-19 pandemic, human social activities have been dramatically changed from the before. Especially, long-term reduced contact with each other during the pandemic resulted in increased or season-sifted epidemic of many infectious diseases thereafter. How do the results in this model reflect the change of human social activity?

The reviewer raises an interesting hypothesis. Indeed, social distancing dramatically changed the circulation of many contagious pathogens. In the NHP facilities, great lengths are taken to protect the NHPs from human pathogens. Any time a human is in the same room as an NHP PPE includes face mask and face shield. Thus human-NHP infections are very rare. Thus we do not

believe the COVID pandemic will have influenced any immunology among the NHPs we study. How the pandemic changed immunity in the human population is certainly of incredible interest.

We have added the following paragraph to the discussion to acknowledge this question, and other Reviewers comments.

Line 520 "Another consideration is the considerable stress the animals undergo during the transition from the outdoor-provisioned environment to the indoor environment. The social hierarchy is altered, and the animals are exposed to more human interactions causing significant stress which can also influence immunity. Further research is required to better understand the relationship between immune phenotypes and human social activity, especially since contact between individuals has significantly changed since the COVID-19 pandemic. It is also possible that biological sex may contribute to immunological alterations occurring during environmental changes. Unfortunately, female NHPs are generally rarely used for biomedical research to maintain colony sizes."

Reviewer #3 (Remarks to the Author):

General
A. Smith et al. analyzed the effects of dewilding young rhesus macaques on hematological, immunological, and microbiological parameters. The subject is of interest because it has direct impacts on preclinical research and the welfare of animals, as well as from a translational perspective. Changes in environmental conditions lead to alterations in complete blood count values, such as a decrease in neutrophil count and an increase in lymphocyte count. Animals housed in laboratory settings exhibited an increase in CD4+ T central memory (T CM) and effector memory (T EM) cells, accompanied by a decrease in naïve CD4+ T cells. Overall, T cells are more activated, with a shift toward a Th1 response against antigens. This is associated with changes in fungal and bacterial microbiota as exposure to environmental factors is reduced in laboratory conditions. Finally, they analyzed the response to measles vaccination in relation to the duration of quarantine and found reduced responsiveness when animals were vaccinated early in the dewilding process. The manuscript is very clear and well written, with nice figures. The methodology is clear and documented. The scheme summarizing the results is also appreciated.

Major points
The project may have been conducted in the Primate Biomedical Facility and may have taken advantage of the ongoing quarantine for specific projects. So, I understand that the individuals chosen depend on the animals available and that some analyses are difficult to run or re-run.

Here are comments or questions that need to be addressed:

The study is based on the concepts of dewilding and rewilding, but it lacks a clear definition within the context of this research. To better understand environmental factors, it would be helpful to describe animal housing in "wild" conditions. For example, is it an outdoor enclosure where young animals (1 or 2 years old) live with adults in a larger group? Are the animals born in the facility, or have they been moved to outdoor enclosures after weaning and transportation from another breeding facility? The weaning age of animals could also have an impact on stress and immunological factors. This suggests that the young rhesus macaques are not in contact with adults, which could have social/sanitary/microbiological impacts.

The reviewer raises several important points. The animals are within the outdoor provisioned environment until they are processed for biomedical research. They are born within the outdoor enclosure and are not processed for biomedical research until after they have been weaned (usually between 9-12 months). Younger and older animals are all located within the outdoor enclosure where normal social structures exist. We completely agree with the reviewer that stress can dramatical influence immunity. The animals have significant stress once processed for biomedical research, when they are moved from the outdoor environment to an indoor, caged, environment. We agree that some of our findings could be influenced by the stress the animals encounter during the transfer. The AGM we study (mentioned above) were born in captivity and have less stress associated with processing. We have significantly revised the Discussion and now discuss many of these limitations in the revised manuscript (see above and below) and thank the Reviewer for his comments.

The second point is that the animals used in the study are still young macaques (1-2 years old), and studies have shown changes in hematological and immunological parameters during the first years of life. (doi: 10.3402/pba.v2i0.18052 , doi: 10.1007/s11357-023-00962-8 , https://doi.org/10.3389/fped.2020.00388)
In the current study, there is no "wild" control group, which could have been used to monitor these changes during quarantine. Therefore, it remains difficult to determine if the observed changes are partly related to animal aging. I also found it challenging to compare CBC values with AGM, as I have never experienced environmental changes, even though the dynamics might be similar; these two species originate from different geographical locations. Another limitation is that only male macaques were used, which may conceal other variations or dynamics present in female macaques.

The reviewer raises an important point. Indeed, Reviewer 1 raised the same point regarding the AGM comparison. We've addressed this point above. In our facility, females are typically only employed if needed to study female reproduction or vaginal microbiome, given the need to keep most females behind to maintain the colony. We have added text to the revised manuscript to discuss this limitation to the studies.

Line 525: "It is also possible that biological sex may contribute to immunological alterations occurring during environmental changes. Unfortunately, female NHPs in our facility are generally rarely used for biomedical research in order to maintain the colony sizes."

The third point is that environmental factors are attributed to the immunological parameters, but Stress factors are not discussed. Dewilding is a significant sociological shift, as animals transition from a large group to a pair. Even in young animals, social status is changed and may also affect immune parameters (DOI: 10.1126/science.aah3580). Therefore, this point should be discussed, as the only change in social hierarchy has been shown to impact TLR4 signaling and pro-inflammatory responses.

We agree with the Reviewer and this point is now further discussed in the revised Discussion the revised manuscript.

Line 520: "Another consideration is the considerable stress the animals undergo during the transition from the outdoor-provisioned environment to the indoor environment. The social hierarchy is altered, and the animals are exposed to more human interactions causing significant stress which can also influence immunity. Further research is required to better understand the relationship between immune phenotypes and human social activity, especially since contact between individuals has significantly changed since the COVID-19 pandemic."

Minor and detailed comments:
Line 245: As mentioned before, using AGM as wild controls is questionable but might be the only wild animals available in the facility. The comparison should be made with caution.

We agree and have now discussed this limitation above (as noted by another Reviewer) and in a new paragraph of the Discussion of the revised manuscript.

Line 450: "An important limitation of our study is that we lacked age-matched wild controls in our analysis because this is logistically impossible. While we found that reduction of neutrophils and expansion of lymphocytes did not occur in age-matched African green monkeys (AGMs) that were bred in captivity and had not undergone environmental changes, AGMs differ substantially from rhesus macaques in T cell development. A more appropriate control would have been either longitudinal analysis of rhesus macaques that were age matched, born in captivity, and had not undergone environmental changes, or longitudinal analysis of rhesus macaques that were maintained in the provisioned outdoor environment. However, it is not feasible to obtain such samples. That AGM bred in captivity did not manifest reduced neutrophils during a similar age window is consistent with our observations in rhesus being attributed to the dewilding. While the AGM are natural hosts for SIVagm and their CD4 T cells down-regulate CD4 with peripheral immunological experience, these do not occur during early life[37]. Moreover, we've not seen hematological differences between lymphocyte, monocyte, and

granulocyte counts between young AGM and RM. Thus, we believe longitudinal analysis of AGM born in captivity are a reasonable control for our “dewilded” rhesus macaques.

Line 248: Parentheses missing.

This has been corrected.

Line 249: Do you have an idea of the parasite/helminth circulation within “wild” animals in the facility? Variation of eosinophils is quite interesting, and your conclusion makes sense but is difficult to prove.

As suggested by the Reviewer, we examined fecal samples from animals in cohort 2 for helminth parasites. Samples from cohort 1 were no longer available. Indeed, helminth parasite burden was usually high in this cohort and 100% of the animals were positive for *Trichuris* parasites and 59% (N = 13) were positive for *Strongyloides*. Historically, our serological analysis has indicated that approximately 25% of the animals were seropositive for *Strongyloides.* We’ve added this information to the revised manuscript.

Line 295: “While data from cohort 1 was unavailable, we determined that 100% of the animals from cohort 2 (N = 22) were positive for *Trichuris* parasites and 59% (N = 13) were positive for *Strongyloides* in fecal samples. Serology analysis from the past indicated that 25.5% (N = 12/47) of analyzed rhesus macaques were seropositive for *Strongyloides*.”

Line 250: The number of cytokines and stimulants used in this part is quite interesting and impressive and could give a nice idea of the PBMC responses. How you selected each antigen would be of added value to better under the strategy used. Only stimulation with CA and BV showed differences in terms of cytokine PMC response. The reduction of IL-17 production with CA stimulation (and SA?), the increase of IL-12p40 secretion with BV, and the increase of CXCL10 (SA and CP), TNF-α (PA) support a shift toward a TH1 response after quarantine. I think citing also that CP, SA, and PA support your finding in this part would be a plus. These bacteria are not cited in the results part, whereas they reinforce the observations.

We agree with the Reviewer that there are other stimulation conditions that were significant that we did not highlight effectively. We have now revised and updated Figure S2 to provide a more complete picture of which cytokines and stimulants are significant. As suggested by the Reviewer, we have also revised the Results section, as below:

Line 308: “Additionally, other Type 1-associated cytokines including CXCL10 (in response to SA and CP), TNF-α (in response to PA), and IFN-γ (in response to BS) were increased following dewilding, suggesting a shift toward a Type 1 response after quarantine. Type 2-associated cytokines were not altered by dewilding (Figure S2).”

Supplementary Figure 2 is very small; a bigger version would be appreciated.

As suggested by Reviewer 1, we have simplified Figure S2 as a heatmap that captures p-values and FDR adjusted p-values to improve visualization and interpretation.

Line 260: In the ICS experiment showing a reduction of IL17+ and an increase of IFNg in CD4+ T cells, it is not clear to me which antigen was used to stimulate the cells. Please specify which antigens you used, as you used several.

For intracellular cytokine staining, the cells were stimulated with PMA and Ionomycin and not with specific antigens. We apologize that this was unclear and have updated the Results and Materials and Methods.

Line 312: "We next examined the capacity of CD4+ and CD8+ T cells to produce cytokines after PMA and ionomycin stimulation, by intracellular cytokine staining of peripheral blood mononuclear cells (PBMCs) (Figures S3-4)."

Line 268: Fecal samples were not taken by rectal swab but by feces collection in cages, if I understood well. This method may be associated with environmental contamination, which, in itself, differs between "wild" and laboratory conditions. Do you think this could impact your microbiota analysis?

The Reviewer raises an interesting idea and is correct, as the fecal samples were obtained from cage feces. However, the concentration of bacterial DNA within feces is approximately 100 billion DNA copies per gram of feces. The concentration within soil ranges from 100 million to 10 billion DNA copies per gram. Thus, we do not believe environmental contamination is a major contributor to our analyses. Indeed, we have previously explored the composition of the microbiome in animals as they transition from the provisioned outdoor environment (PMID 36475838) and we did not find high levels of soil bacteria. Since we consistently only find typical GI tract microbes, we don't think that environmental contamination from feces collection from the cage significantly impacts our analysis.

Line 287: The bone marrow analysis seems to be performed on frozen cells. The freezing and thawing of blood and bone marrow can result in partial or complete depletion of granulocytes. The fact that results from BM do not reflect those from the blood might be due to a selective depletion of granulocytes. On supplementary figures 9 and 10, I'm surprised to observe so few FSC high SCC high granulocytes, whereas macaque bone marrow is composed of 70 to 80% granulocytes. Only the stem cells can be cryopreserved and analyzed here. I would analyze the phenotype of immature and mature neutrophils with caution, as the freezing process significantly alters these populations. Additionally, the classification used for CD11b and CD87 in neutrophils is unfamiliar to me. Could you cite the paper supporting this classification?

We completely agree with the Reviewer that the freezing and thawing of bone marrow cells for analysis can severely affect granulocyte populations and that we should interpret differences between the bone marrow and peripheral blood with caution. Hence, we have significantly changed the paragraph in the Discussion addressing these results. Here is the new paragraph:

Line 475: "However, the changes observed in peripheral blood during dewilding were not mirrored in the BM. In peripheral blood, dewilding led to reduced neutrophils and increased lymphocytes, whereas in the BM, we found increased granulocytes and reduced lymphocytes. An important limitation of this observation is that freezing and thawing of bone marrow cells could more severely affect granulocyte populations than other cell types. Alternatively, during dewilding we observed a shift towards a proinflammatory T_H1 response, producing proinflammatory cytokines TNF-α and IFN-γ, which could promote lymphocyte egress from the BM, allowing granulocyte precursors to expand into the vacated space and undergo maturation. This dynamic would be consistent with our observations of increased expression of neutrophil maturation markers, such as CD11b and CD10[36,37], in BM granulocytes, alongside accumulating $CD87^{hi}$ mature neutrophils during dewilding."

The paper on CD11b and CD87 is: Weisgrau KL, Vosler LJ, Pomplun NL, Hayes JM, Simmons HA, Friedrichs KR, Rakasz EG. Neutrophil progenitor populations of rhesus macaques. *J Leukoc Biol.* 2019 Jan;105(1):113-121. doi: 10.1002/JLB.1TA1117-431RR. Epub 2018 Nov 5. PMID: 30395351; PMCID: PMC6398341. We have now included this in the citations.

Line 345: The vaccine protocol used here seems to rely on only one shot of the measles vaccine. The vaccine used and the immunization protocol need to be described. The vaccine protocol in macaques typically involves two shots (administered 3 to 6 months apart) of MMR and is associated with seroconversion rates of 90-95%. So, if such a protocol has been used here, I'm surprised by the low level of seroconversion. Otherwise, if only one shot has been used, I'm not surprised, but this is not the standard protocol. The conclusion on these results should be cautiously revised in this regard.

The Reviewer raises an important point. Indeed, even when the titers of antibody induced was low, all the animals did seroconvert. Instead of referring to individuals as responders and non-responders, we now refer to them as high responders and low responders.

In our NHP facility the standard of care is to provide one dose of MMR and then assess seroconversion. If any particular animals do not seroconvert (which is rare and not seen in our cohort), a second dose of MMR is provided. We have added this information to the methods section of our revised manuscript.

The ELISA we used to measure antibodies against measles was not quantitative. To approximate levels of antibodies made after vaccination, we reported optical density values. We've reclassified the animals based on "high" and "low" responders, and hope this clarifies the issue.

Additionally, as noted by other Reviewers, the differences between early and late vaccinations are not statistically significant and we have significantly toned down the conclusions and discussion of this result.

Line 437: The Authors discussed the possibility that infection with RhCMV may influence the immune response observed in the study. This is a really good point, as these young animals are likely to get infected. Is it possible to show data on the seroconversion of macaques against CMV?

The CMV seropositivity rate of the animals in our facility is 100%. We do not know the CMV serology status of the animals within the provisioned outdoor environment. Given the low seroprevalence of CMV among human infants, we believe the animals acquire CMV once they enter the indoor facility. We've added this information to the revised manuscript.

Line 526: "One possibility for the heightened T_H1 response observed during dewilding may be infection with rhesus cytomegalovirus (RhCMV), common in captive rhesus macaques[59]. Indeed, all animals within our indoor environment are RhCMV seropositive, however we do not know if the macaques acquire CMV in the process of dewilding."
